# Supplementary material for: Antiproliferative Fatty Acids Isolated from the Polypore Fungus Onnia tomentosa
Source: J Fungi (Basel). 2022 Nov 3;8(11):1163. doi: 10.3390/jof8111163 (PMC9693168; doi:10.3390/jof8111163)
Supplement: Supplementary file 1 [file jof-08-01163-s001.zip › jof-1939606_Supplementary Materials_edited.pdf]

Supplementary Materials

Antiproliferative Fatty Acids Isolated from the Polypore Fungus *Onnia tomentosa*

Hooi Xian Lee [1], Wai Ming Li [1], Jatinder Khatra[1], Zhicheng Xia [2], Oleg Sannikov [2], Yun Ling [2], Haoxuan Zhu [2], and Chow H. Lee [1,*]

[1]Department of Chemistry and Biochemistry, Faculty of Science and Engineering, University of Northern British Columbia, Prince George, BC V2N 4Z9, Canada.

[2]Department of Chemistry, University of British Columbia, Vancouver, BC V6T 1Z1, Canada.

*Corresponding authors: chow.lee@unbc.ca

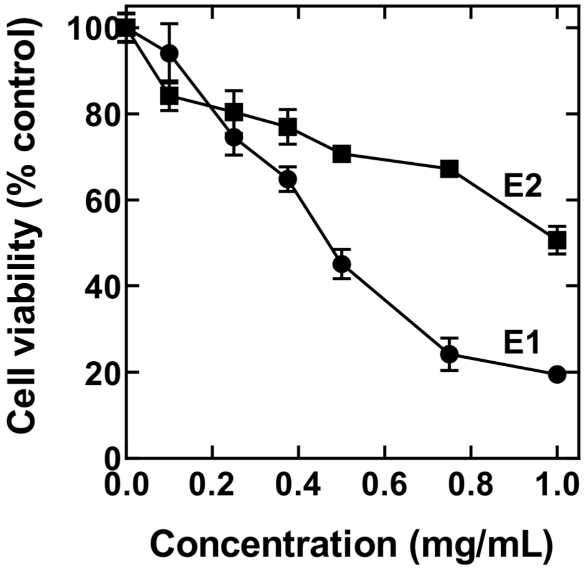

Figure S1. Effects of crude extracts from *O. tomentosa* (CL312) on HeLa cells. Cells were treated with different concentrations of E1 and E2 extracts from *O. tomentosa* for 48 h, followed by cell viability assessment using the MTT assay. The result shown is representative data from three biological replicates (n = 3). Error bars indicate standard deviation (S.D.).

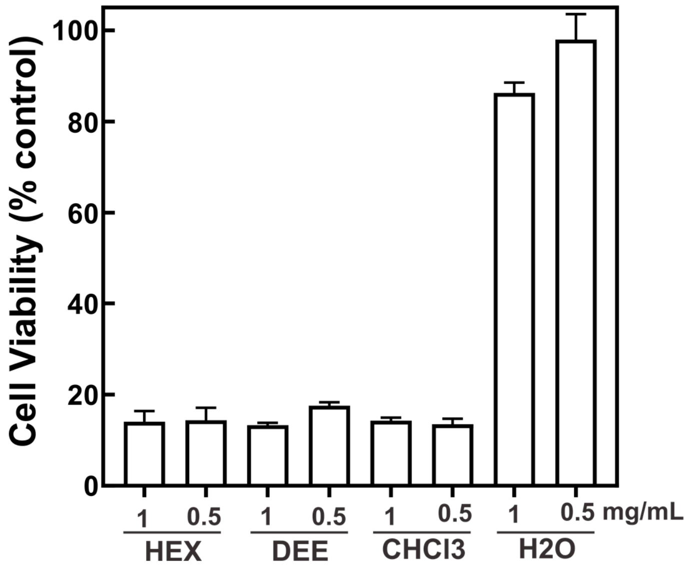

Figure S2. Effects of E1-extracted organic phase separated layers on the proliferation of HeLa cells. Cells were treated with two different concentrations of hexane (HEX), diethyl ether (DEE), chloroform ($CHCl_3$), and water (H_2O) layers for 48 hours, and cell viability was measured using the MTT assay. The result shown is representative data from three biological replicates (n = 3). Error bars indicate standard deviation (S.D.).

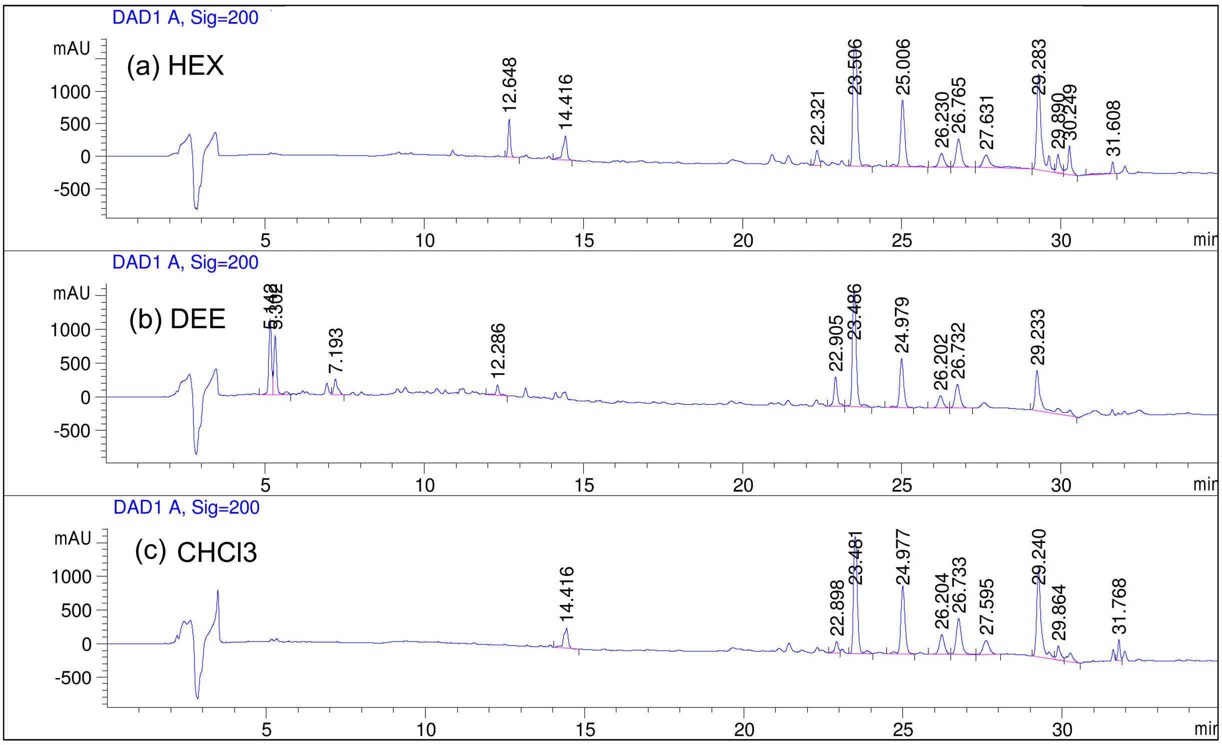

Figure S3. HPLC UV spectrum at λ= 200 nm. Comparing the detected peaks amongst the phase-separated organic layers: (a) hexane (HEX); (b) diethyl ether (DEE); (c) chloroform ($CHCl_3$). Analysis was done using a phenyl-hexyl column (4.6 mm × 250 mm × 5μm) with a gradient mobile phase composed of an H_2O solution of 0.1 % formic acid (solvent A) and CH_3CN containing 0.1% formic acid (solvent B); flow rate of 1 mL/min. The gradient elution program was set as follows: 0 min (25% B), 5 min (35% B), 12 min (70% B), 17 min (70 % B), 20 min (85% B), 25 min (85% B), 28 min (100% B), and 35 min (100% B).

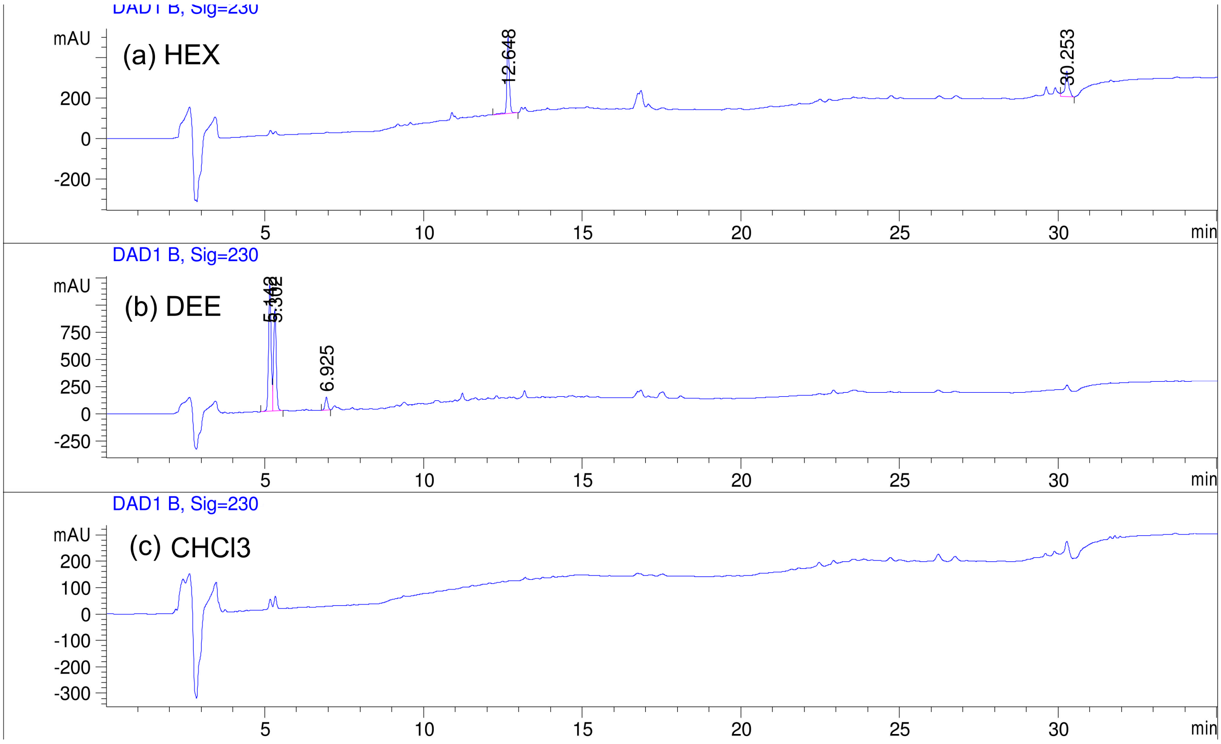

Figure S4. HPLC UV spectrum at λ= 230 nm. Comparing the detected peaks amongst the phase-separated organic layers: (a) hexane (HEX); (b) diethyl ether (DEE); (c) chloroform ($CHCl_3$). The chromatographic system (column and solvents) used was as those described in Figure S3.

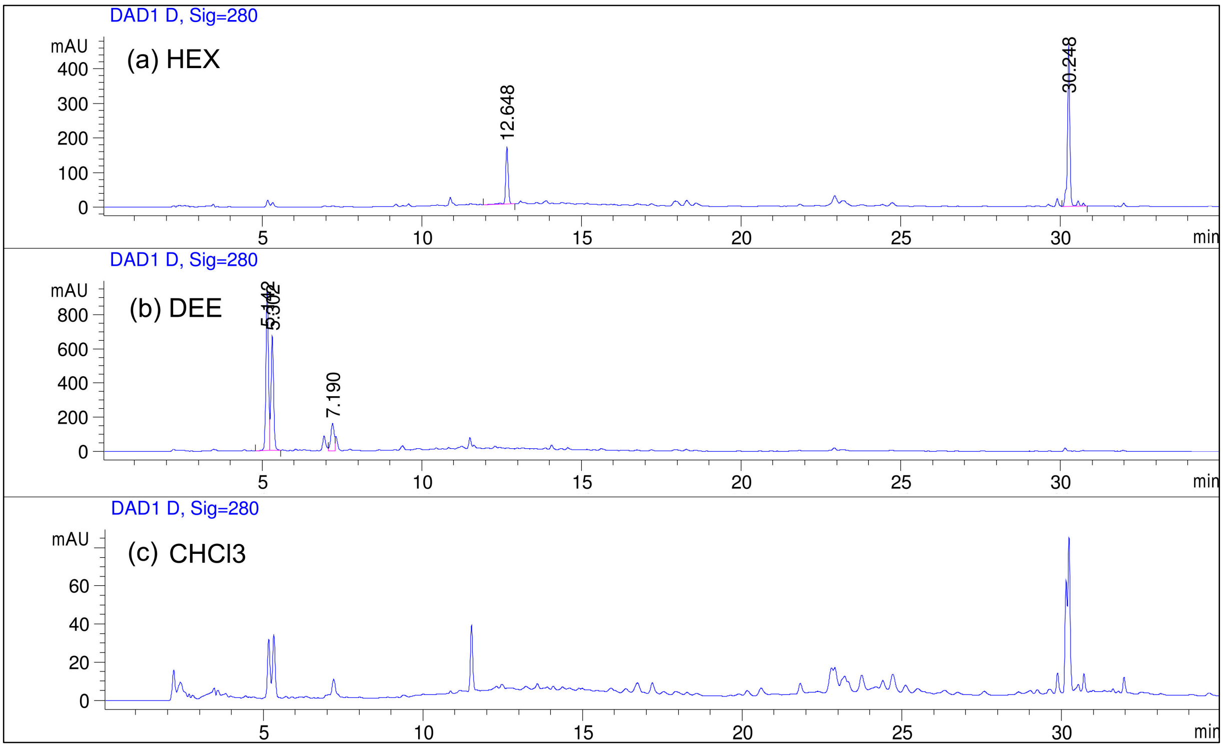

Figure S5. HPLC UV spectrum at λ= 280 nm. Comparing the detected peaks amongst the phase-separated organic layers: (a) hexane (HEX); (b) diethyl ether (DEE); (c) chloroform ($CHCl_3$). The chromatographic system (column and solvents) used was as those described in Figure S3.

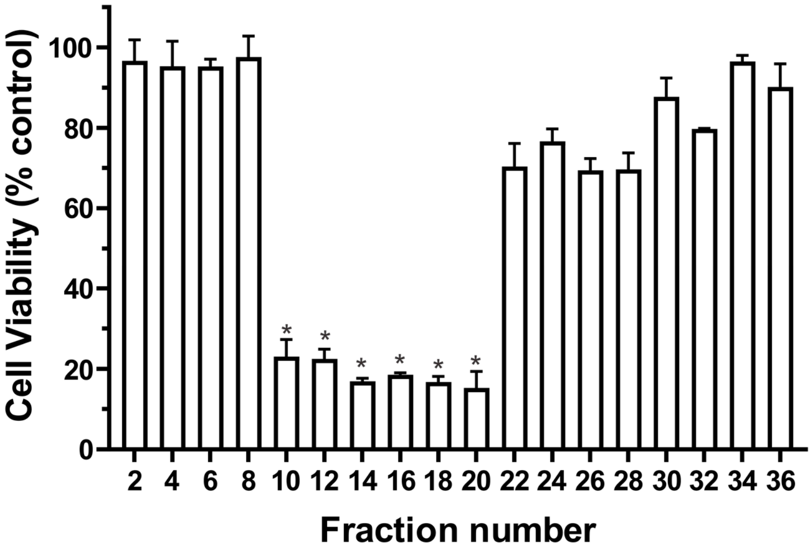

Figure S6. Effects of post-Sephadex LH-20 fractions on the proliferation of HeLa cells. Hexane layer extract was run on Sephadex LH-20 column and fractions were collected as described in the Materials and Methods. Two µL of each fraction was added to HeLa cells in 96-well plates. Cell viability was assessed using the MTT assay. The result shown is representative data from three biological replicates (n = 3). Error bars indicate standard deviation (S.D.). *Indicated the active fractions.

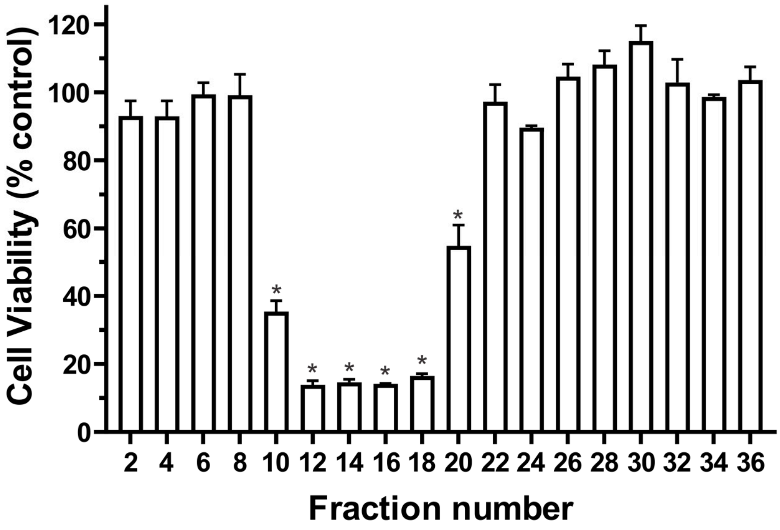

Figure S7. Effects of post-Sephadex LH-20 fractions on the proliferation of HeLa cells. Diethyl ether layer extract was run on Sephadex LH-20 column and fractions were collected as described in the Materials and Methods. Two µL of each fraction was added to HeLa cells in 96-well plates. Cell viability was assessed using the MTT assay. The result shown is representative data from three biological replicates (n = 3). Error bars indicate standard deviation (S.D.). *Indicated the active fractions.

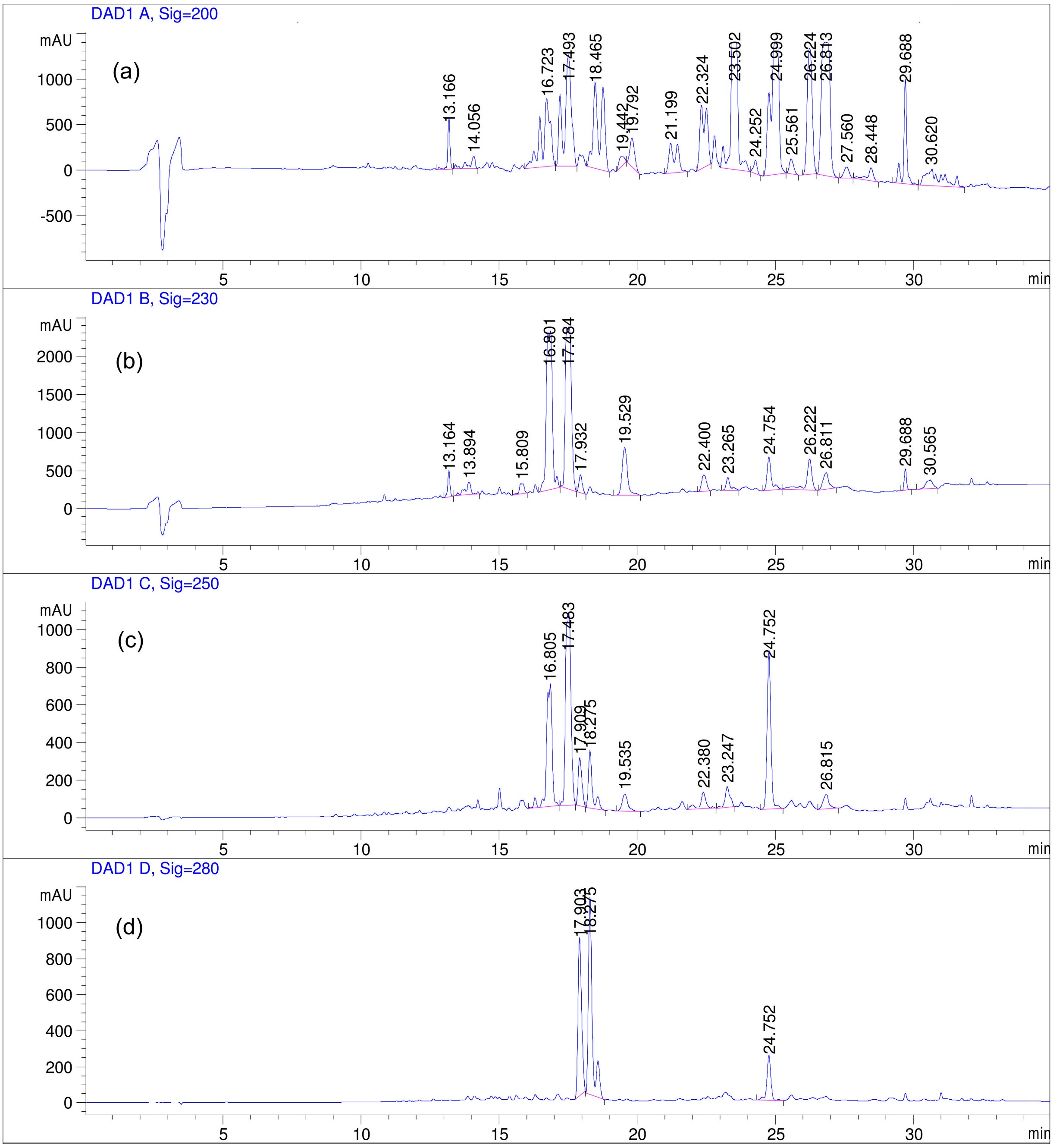

Figure S8. HPLC analyses of HEX layer post-Sephadex LH-20 fractionation. UV spectrum showing peaks detected at the different wavelengths: (a) 200nm; (b) 230nm; (c) 250nm; (d) 280nm. The chromatographic system (column and solvents) used was as those described in Figure S3.

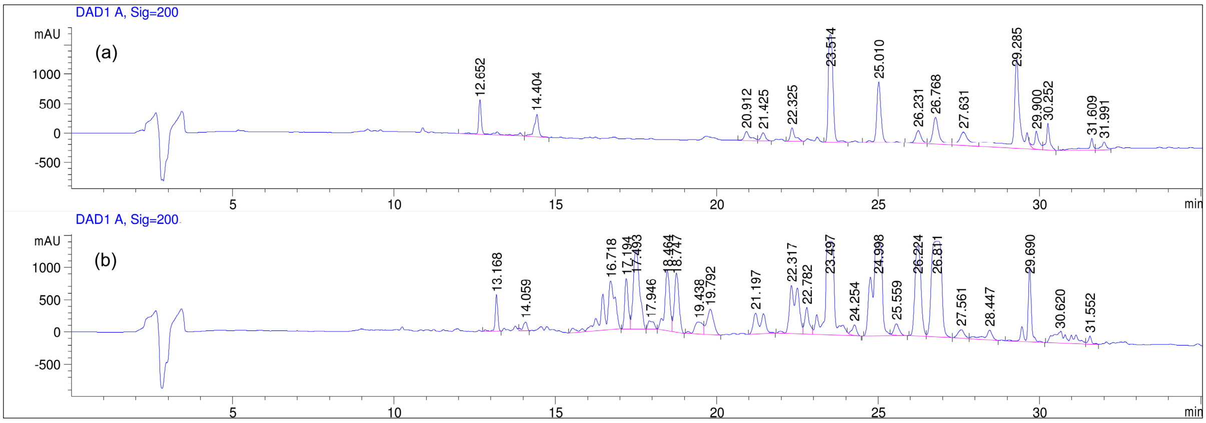

Figure S9. HPLC analyses of the HEX layer before and after Sephadex LH-20 chromatography. UV spectrum showing peaks detected at λ= 200 nm in the HEX layer: (a) before Sephadex LH-20 fractionation; (b) post-Sephadex LH20 fractionation. The chromatographic system (column and solvents) used was as those described in Figure S3.

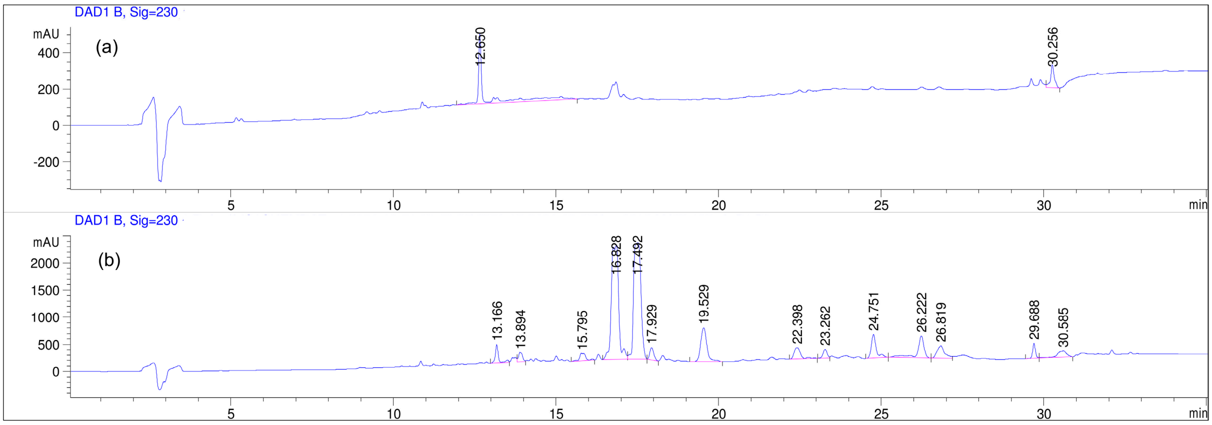

Figure S10. HPLC analyses of the HEX layer before and after Sephadex LH-20 chromatography. UV spectrum showing peaks detected at λ= 230 nm in the HEX layer: (a) before Sephadex LH-20 fractionation; (b) post-Sephadex LH20 fractionation. The chromatographic system (column and solvents) used was as those described in Figure S3.

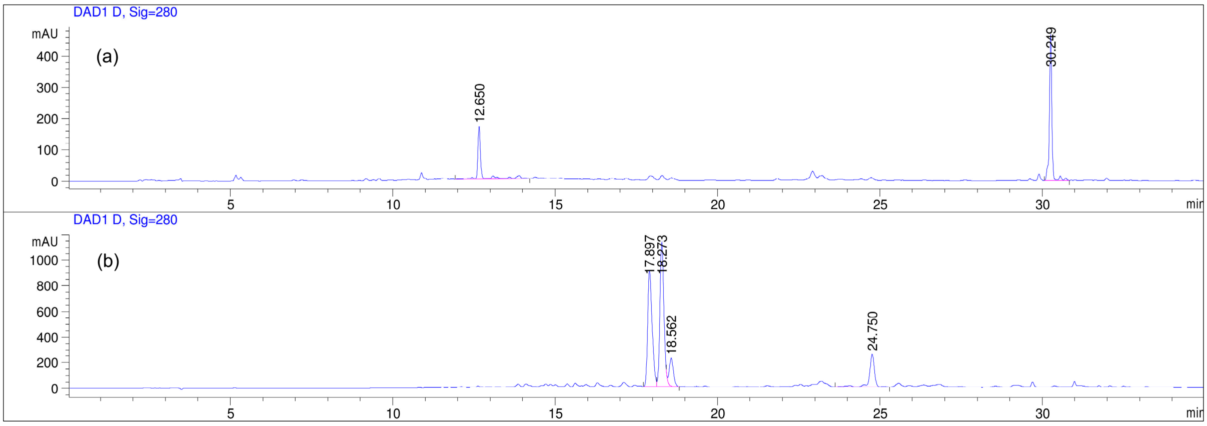

Figure S11. HPLC analyses of the HEX layer before and after Sephadex LH-20 chromatography. UV spectrum showing peaks detected at λ= 280 nm in the HEX layer: (a) before Sephadex LH-20 fractionation; (b) post-Sephadex LH20 fractionation. The chromatographic system (column and solvents) used was as those described in Figure S3.

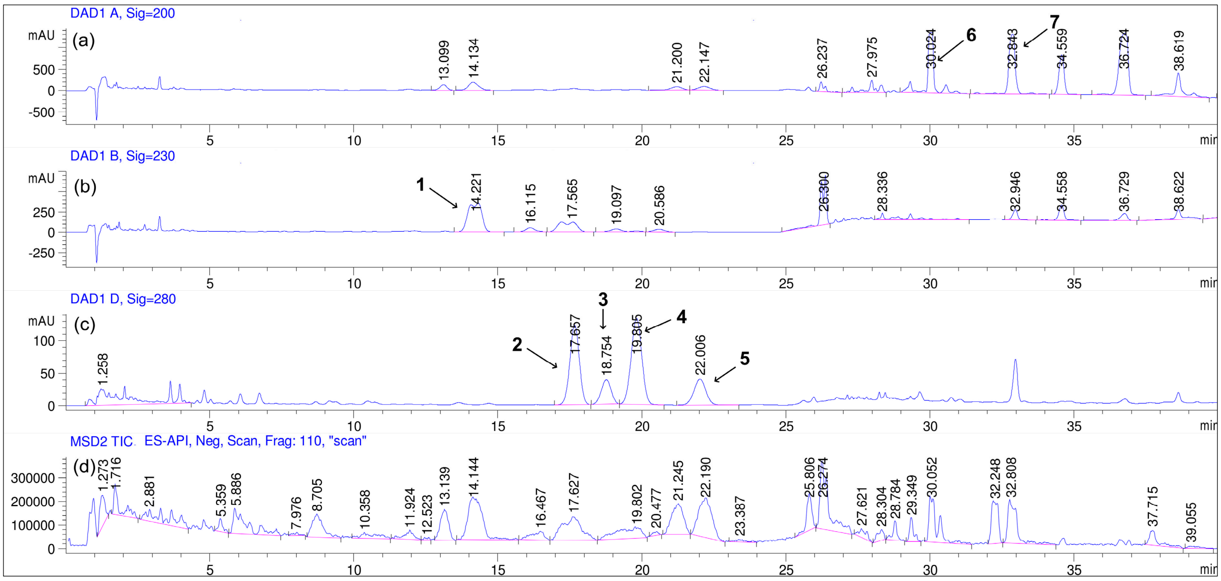

Figure S12. HPLC-MS analyses of antiproliferative fraction collected from Sephadex LH-20 column chromatography. HPLC UV spectrum showing the peaks detected at: (a) 200 nm; (b) 230 nm; (c) 280 nm. (d) HPLC-MS scan spectrum showed peaks detected from *m/z* 100 to 1000. Numbers **1**–**7** were the compounds identified in the active fraction. Analysis was done using a Phenomenex Luna C18 (2) column (4.6 mm × 100 mm × 3µm) with a gradient mobile phase composed of an H_2O solution of 0.1% formic acid (solvent A) and CH_3CN containing 0.1% formic acid (solvent B); flow rate of 1mL/min. The gradient elution program was set as follows: 0 min (52% B), 23 min (52% B), 25 min (90% B), 35 min (90% B), 38 min (100% B), and 40 min (100% B).

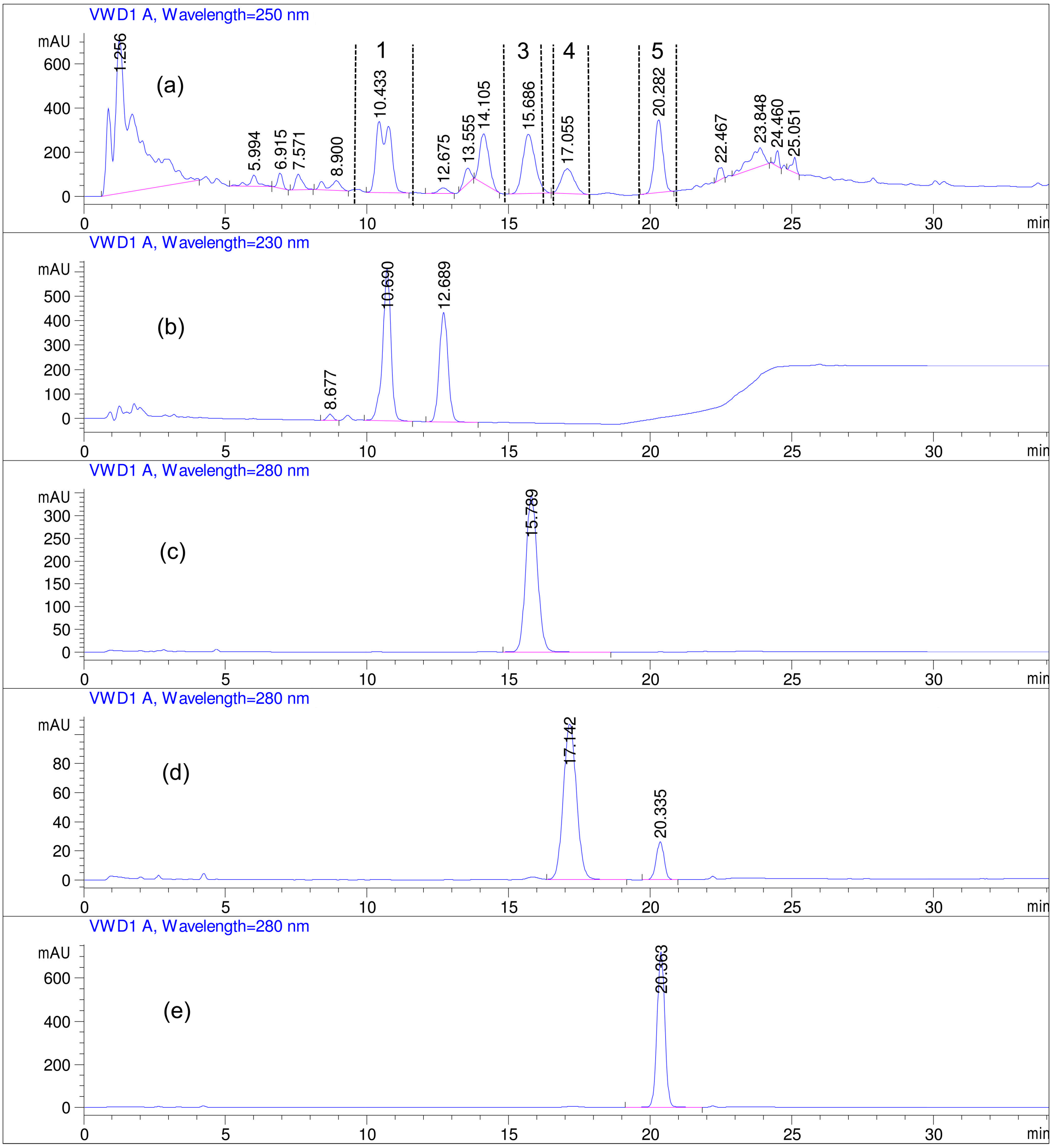

Figure S13. HPLC UV chromatogram showing mixture **1** and compounds **3**–**5** purification process: (a) before HPLC chromatographic purification (the dashed lines represent the collected fraction during the fractionation process); (b) mixture **1** post-HPLC chromatographic purification; (c) compound **3** post-HPLC chromatographic purification; (d) compound **4** post-HPLC chromatographic purification (exists as a mixture with compound **5** detected); (e) compound **5** post-HPLC chromatographic purification. Purification was performed using an Agilent Infinity Lab Poroshell 120 EC-C18 column (4.6 mm × 100 mm × 2.7 µm) with a gradient mobile phase composed of an H_2O solution of 0.1% formic acid (solvent A) and CH_3CN containing 0.1% formic acid (solvent B); flow rate of 1 mL/min. The gradient elution program was set as follows: 0 min (55% B), 17 min (46.5% B), 21 min (70% B), 23 min (100% B), and 35 min (100% B).

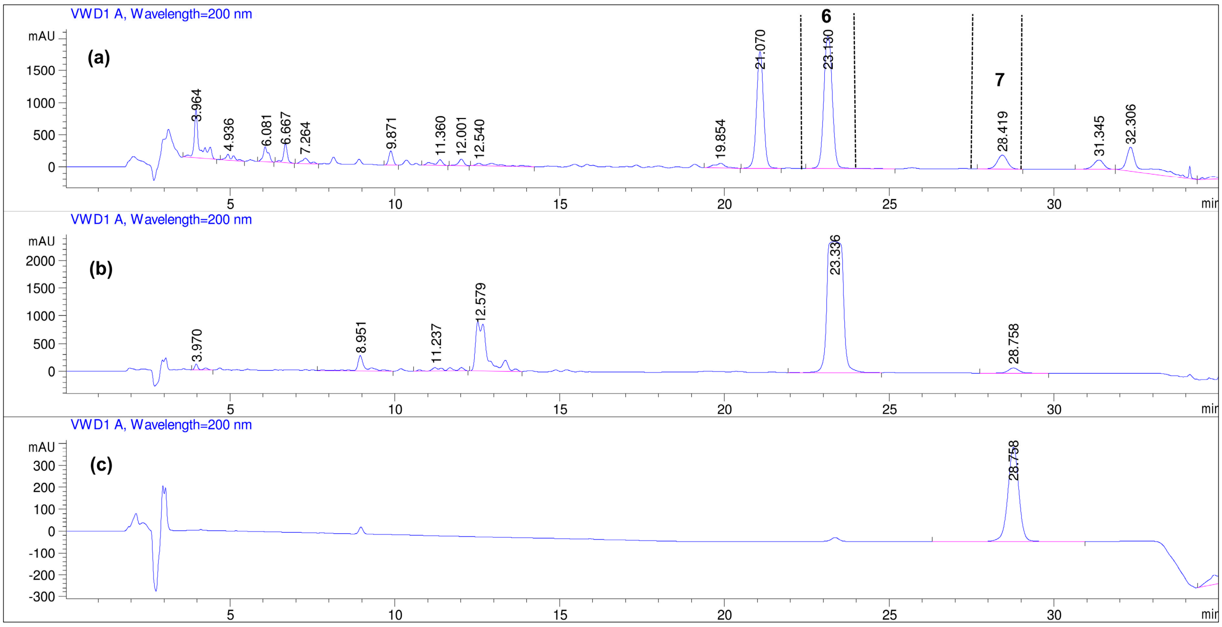

Figure S14. HPLC UV chromatogram showing compounds **6** and **7** purification process: (a) before HPLC chromatographic purification (the dashed lines represent the collected fraction during the fractionation process); (b) compound **6** post-HPLC chromatographic purification (exists as a mixture of compounds); (c) compound **7** post-HPLC chromatographic purification. Purification was performed using a Phenomenex Phenyl-hexyl column (4.6 mm × 250 mm × 5 µm) with a gradient mobile phase composed of an H_2O solution of 0.1% formic acid (solvent A) and CH_3CN containing 0.1% formic acid (solvent B) at a flow rate of 1 mL/min. The mobile phase gradient was set as follows: 0 min (55% B), 15 min (70% B), 31 min (70% B), 32 min (100% B), and 35 min (100% B).

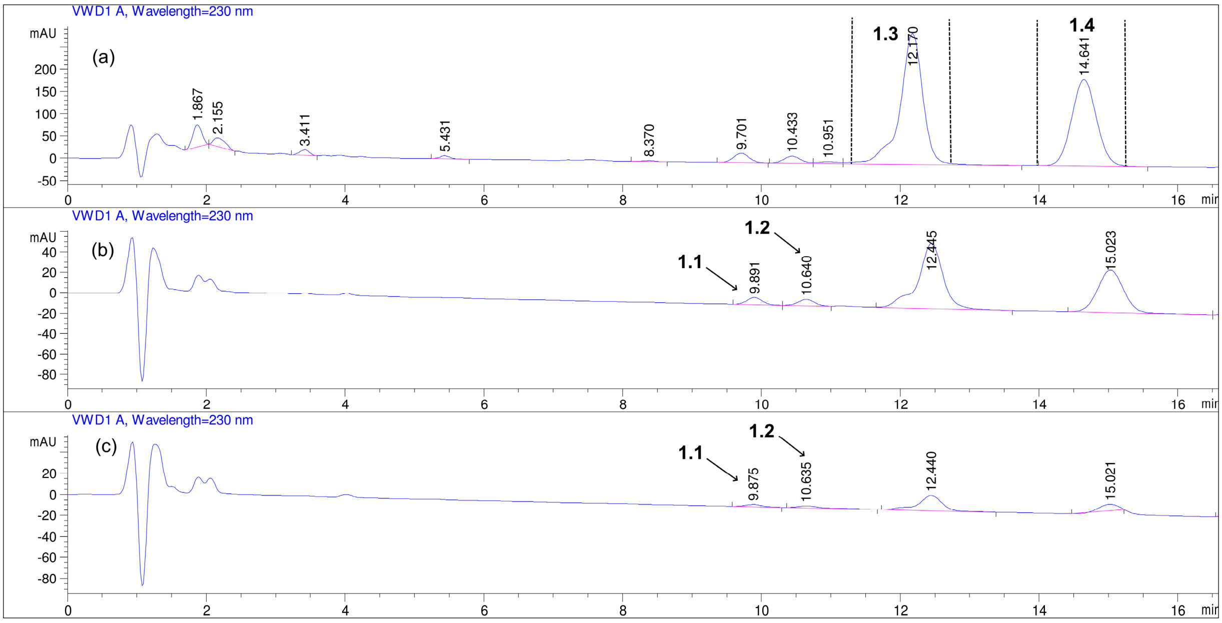

Figure S15. HPLC UV chromatogram showing mixture **1** purification process: (a) before HPLC chromatographic purification of compounds labeled as **1.3** and **1.4** (the dashed lines represent the collected fraction during the fractionation process); (b) compound **1.3** post-HPLC chromatographic purification produced two major (compounds **1.3** and **1.4**) and two minor (compounds **1.1** and **1.2**) peaks. (c) compound **1.4** post-HPLC chromatographic purification produced two major (compounds **1.3** and **1.4**) and two minor (compounds **1.1** and **1.2**) peaks. Purification was performed using an Agilent Infinity Lab Poroshell 120 EC-C18 column (4.6 mm × 100 mm × 2.7 µm) with a gradient mobile phase composed of an H_2O solution of 0.1% formic acid (solvent A) and CH_3CN containing 0.1% formic acid (solvent B); flow rate of 1 mL/min. The gradient elution program was set as follows: 0 min (55% B), 15 min (47.5% B), and 17 min (100% B).

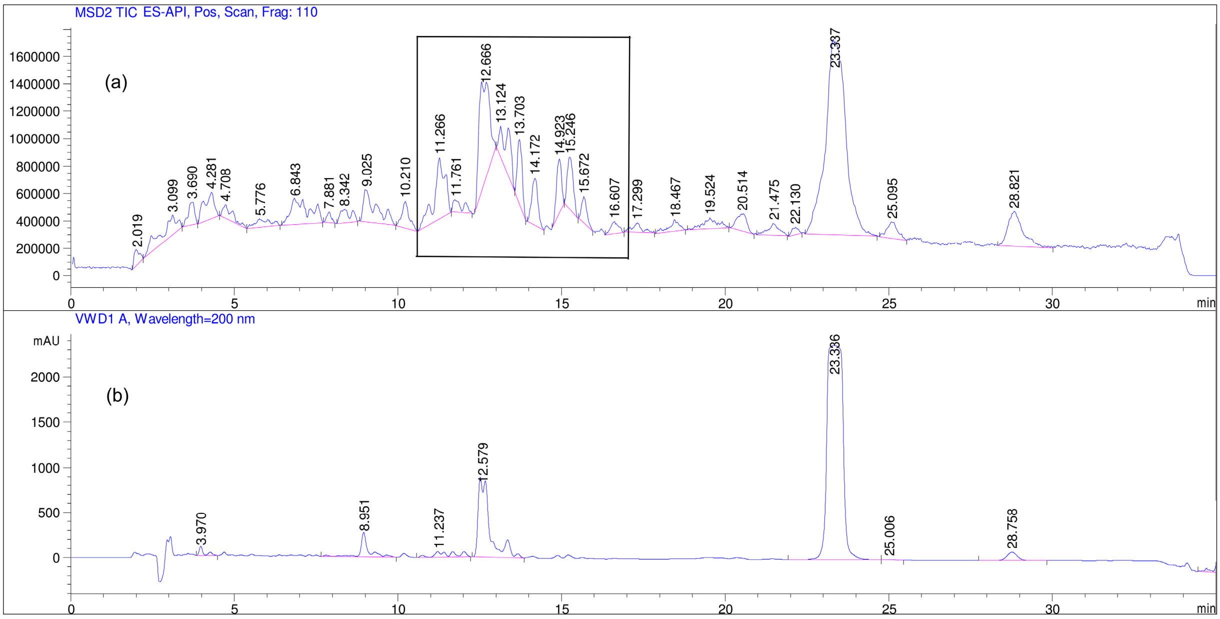

Figure S16. HPLC-LRMS analysis of compound **6**. (a) MS scan spectrum detected from *m/z* 100 to 1000 (the boxed area indicates detected compounds with mass of 294–296 Da); (b) compound **6** post-HPLC purification (exists as a mixture of compounds). The chromatographic system (column and solvents) used was as those described in Figure S14.

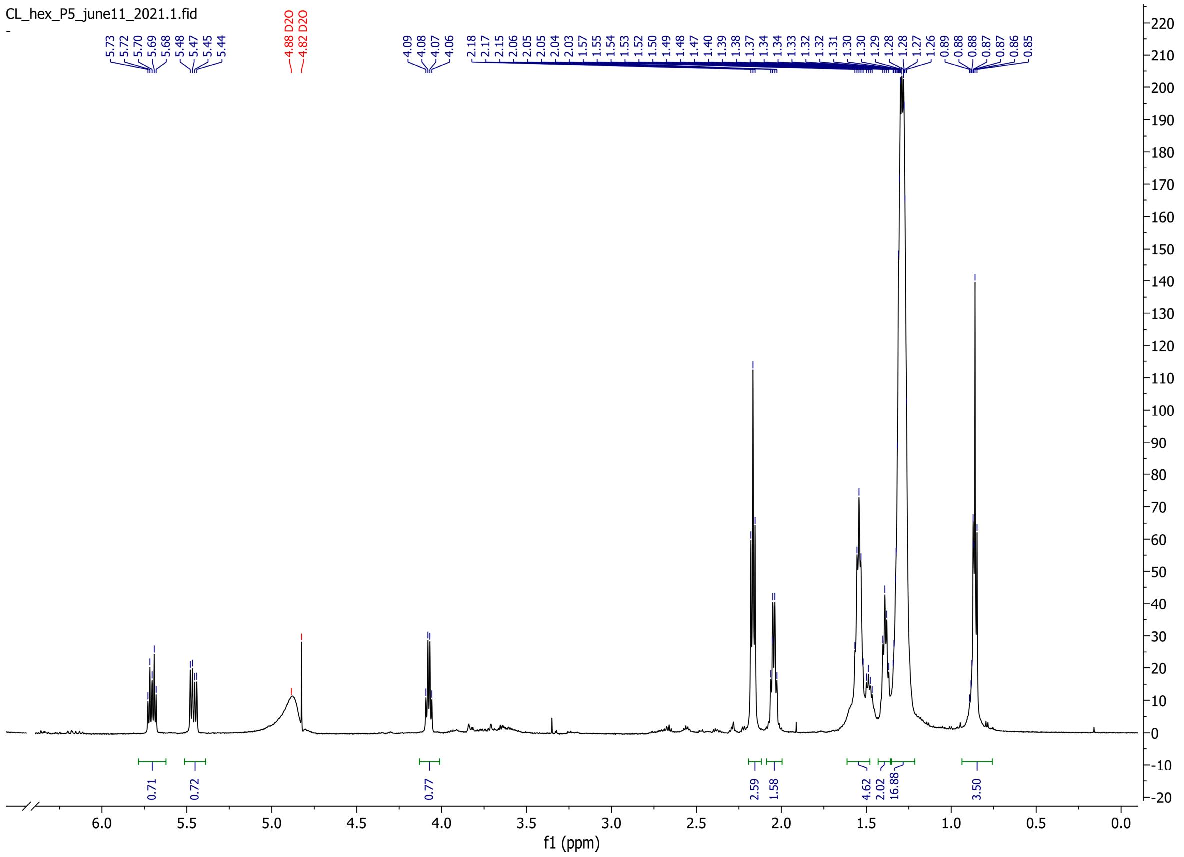

Figure S17. ^{1}H NMR spectrum of **5**. Sample was prepared in D_2O and run at 600 MHz.

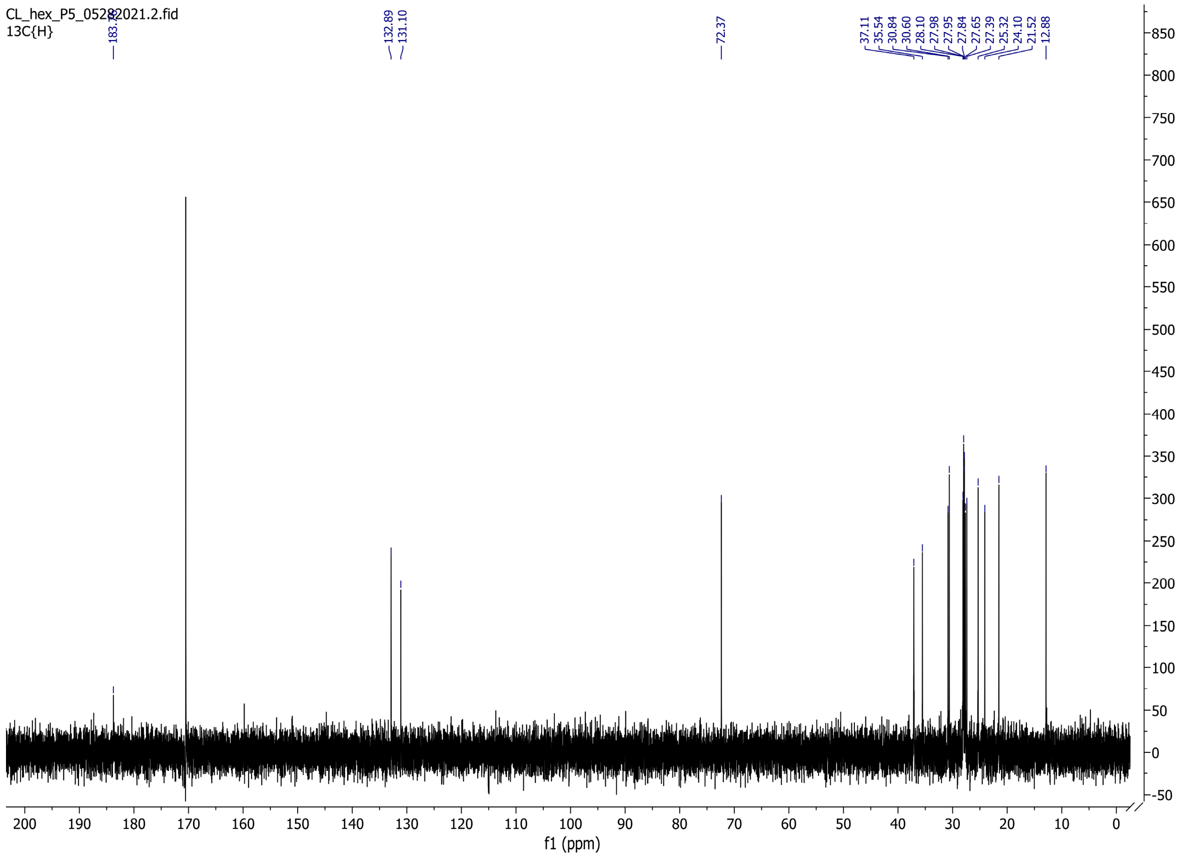

Figure S18. ^{13}C NMR spectrum of **5**. Sample was prepared in D_2O and run at 151 MHz.

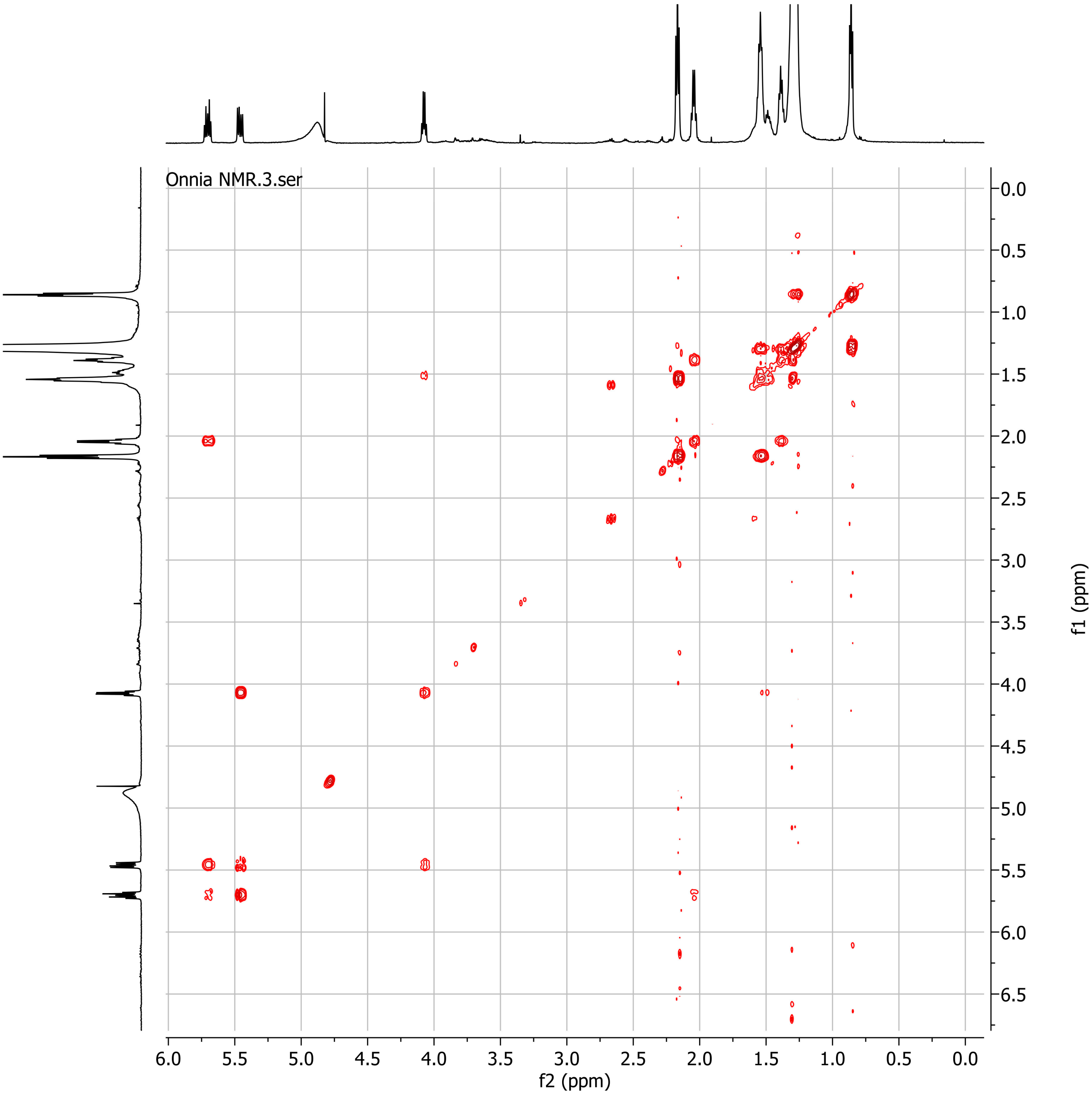

Figure S19. COSY (^{1}H-^{1}H)-2D NMR spectrum of **5**. Sample was prepared in D_2O.

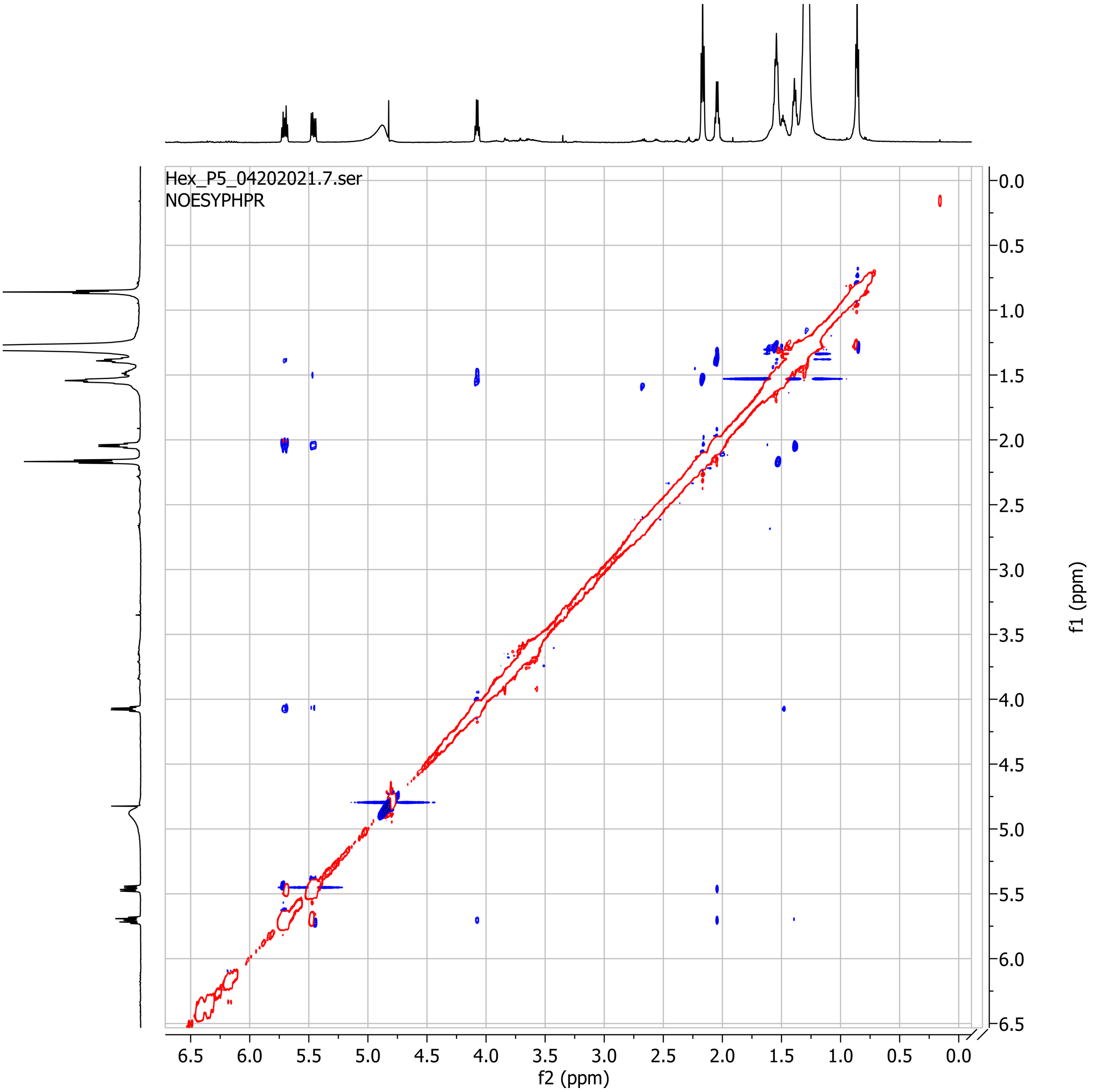

Figure S20. NOESY (^{1}H-^{1}H)-2D NMR spectrum of **5**. Sample was prepared in D_2O.

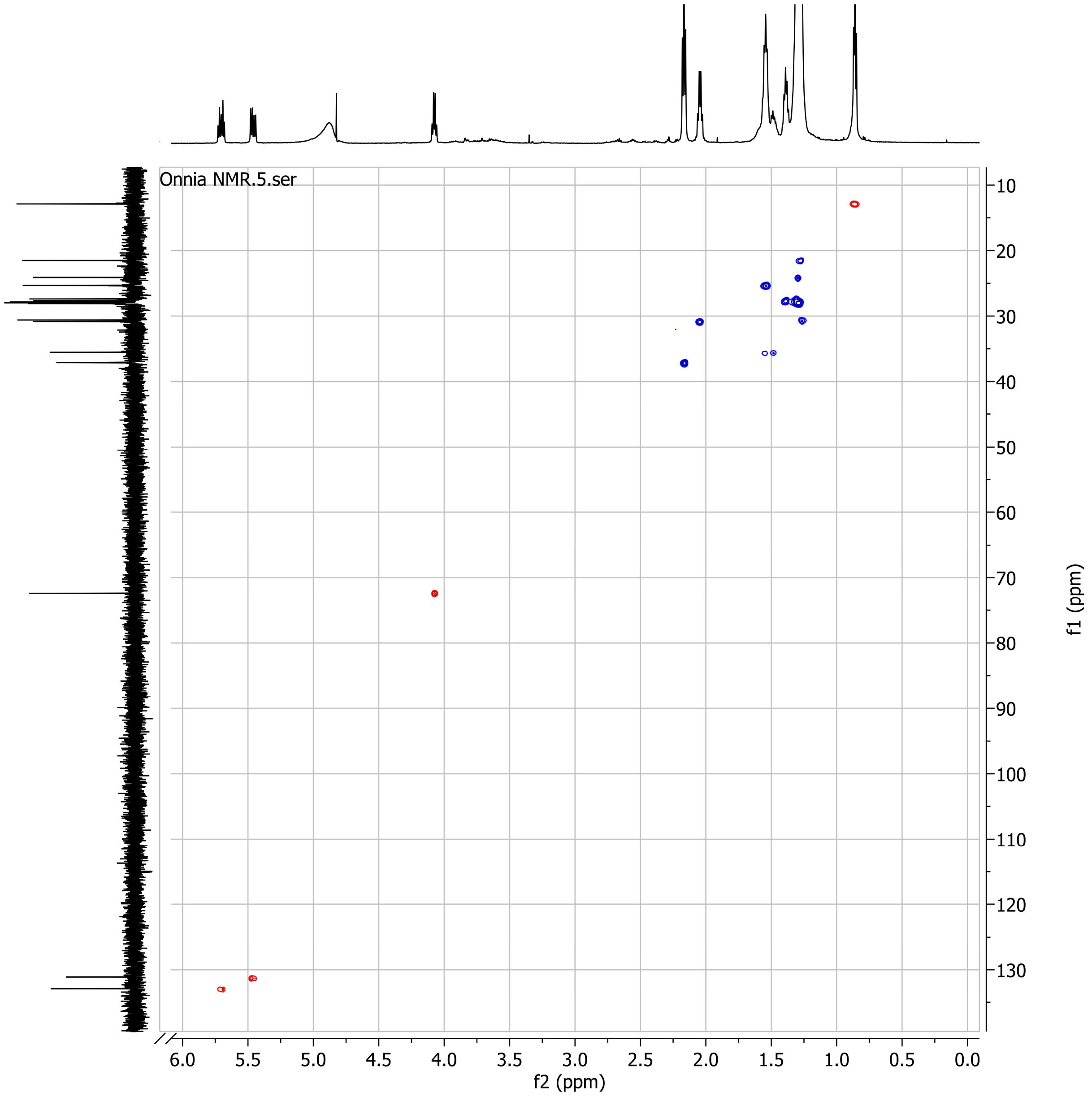

Figure S21. HSQC (^1H-^{13}C)-2D NMR spectrum of **5**. Sample was prepared in D_2O.

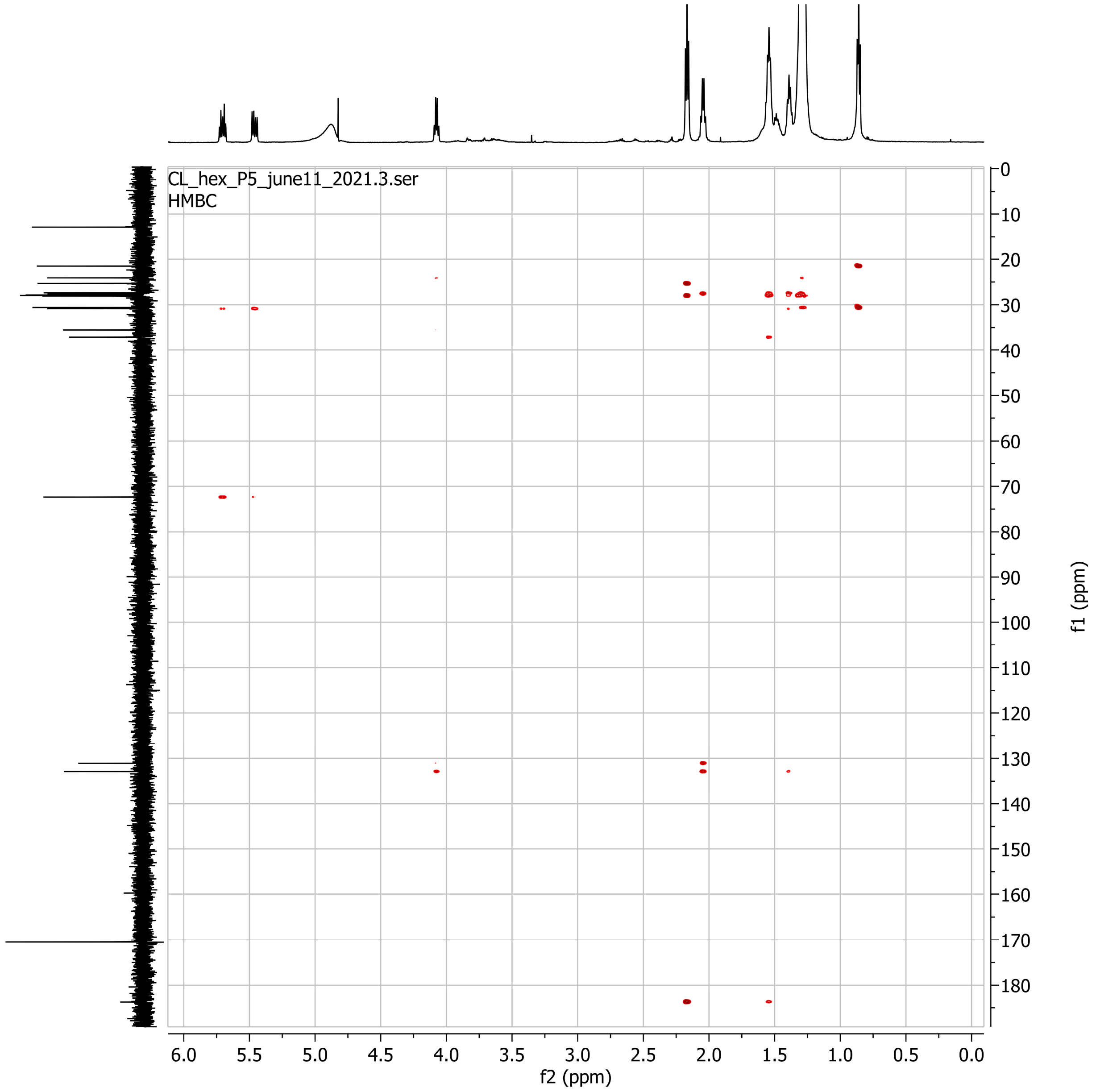

Figure S22. HMBC (^1H-^{13}C)-2D NMR spectrum of **5**. Sample was prepared in D_2O.

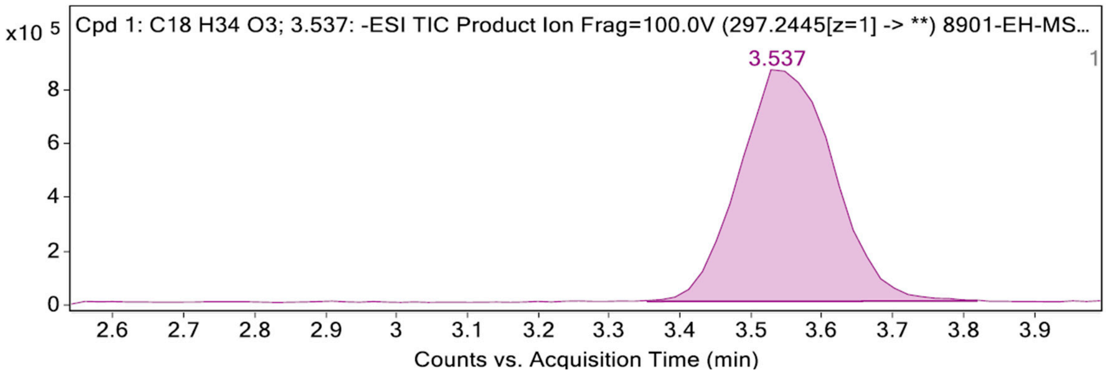

Figure S23. Extracted ion chromatogram (EIC) of compounds **5** isolated from *O. tomentosa*. Analysis was done using an Agilent Zorbax RRHD Eclipse Plus C18 column (2.1 mm × 50 mm × 1.8 µm) with an isocratic mobile phase composed of an H_2O solution of 0.1% formic acid (solvent A) and CH_3CN containing 0.1% formic acid (solvent B); flow rate of 0.15 mL/min. The isocratic elution program was set as follows: 0–5 min (70% B).

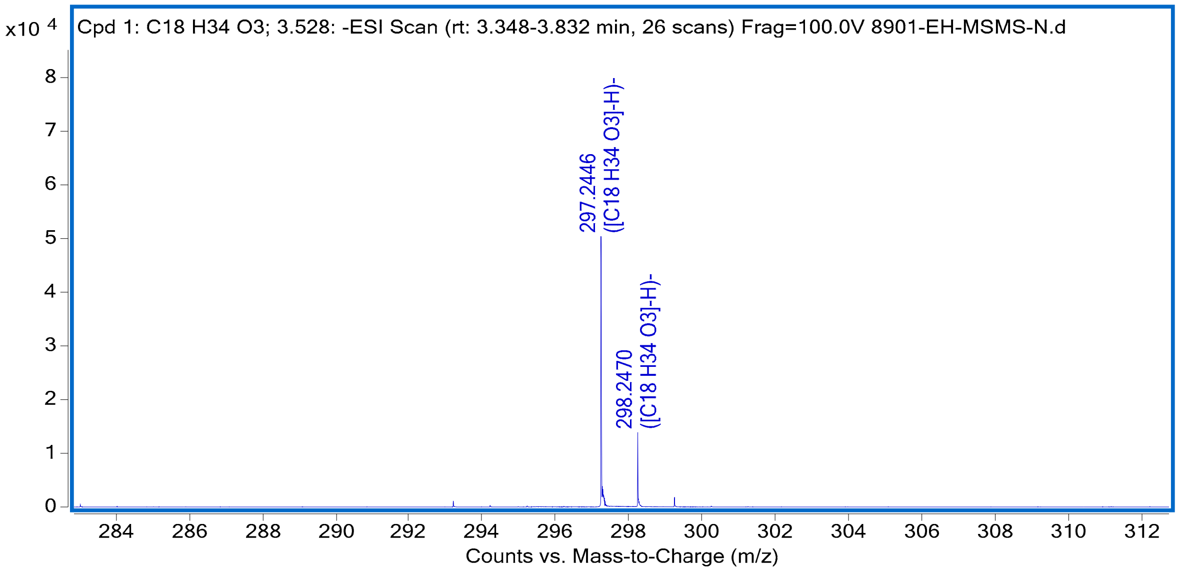

Figure S24. Isotope pattern of compound **5** isolated *O. tomentosa*.

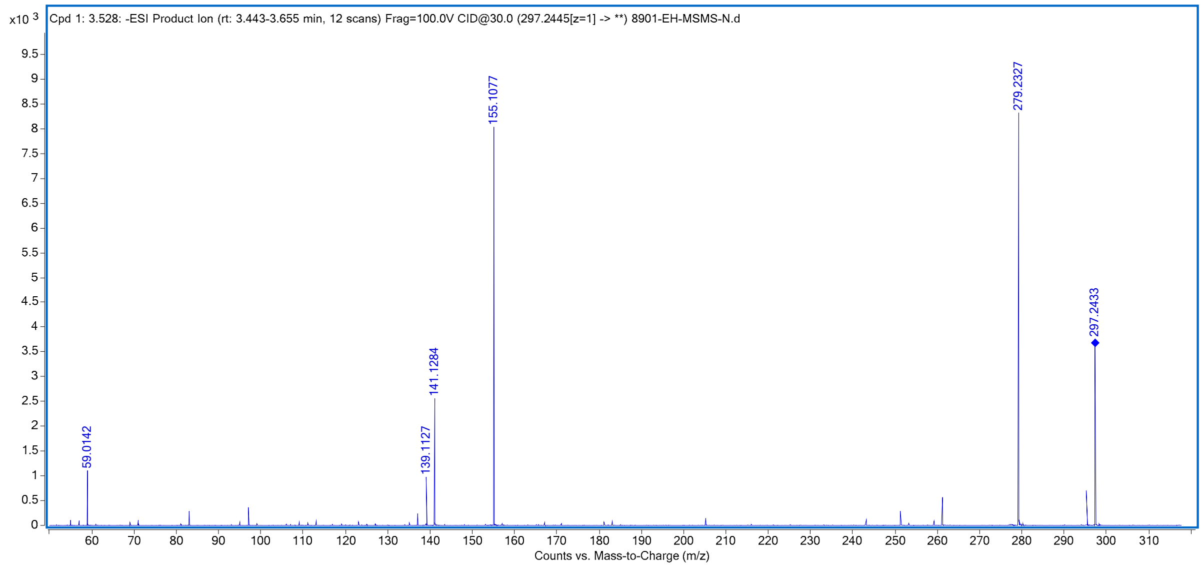

Figure S25. ESI-HRMS/MS spectrum of compound **5** isolated from *O. tomentosa*.

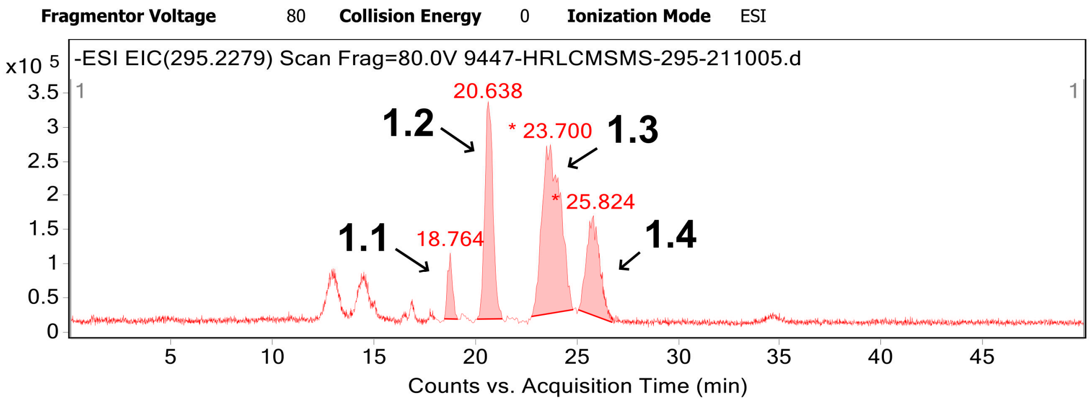

Figure S26. Extracted ion chromatogram (EIC) of compounds **1.1** to **1.4** isolated from *O. tomentosa*. Analysis was done using an Agilent Zorbax RRHD Eclipse Plus C18 column (2.1 mm × 50 mm × 1.8 µm) with an isocratic mobile phase composed of an H_2O solution of 0.1% formic acid (solvent A) and CH_3CN containing 0.1% formic acid (solvent B); flow rate of 0.5 mL/min. The isocratic elution program was set as follows: 0–50 min (48% B).

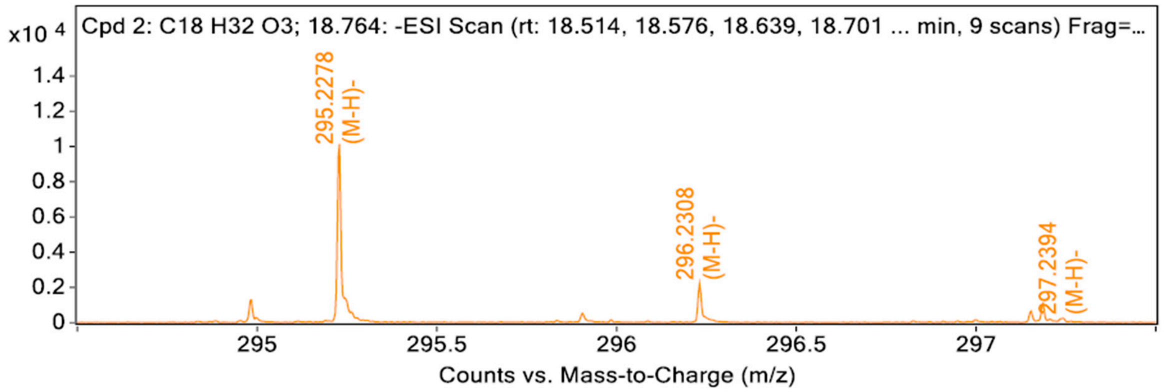

Figure S27. Isotope pattern of compound **1.1** isolated from *O. tomentosa*.

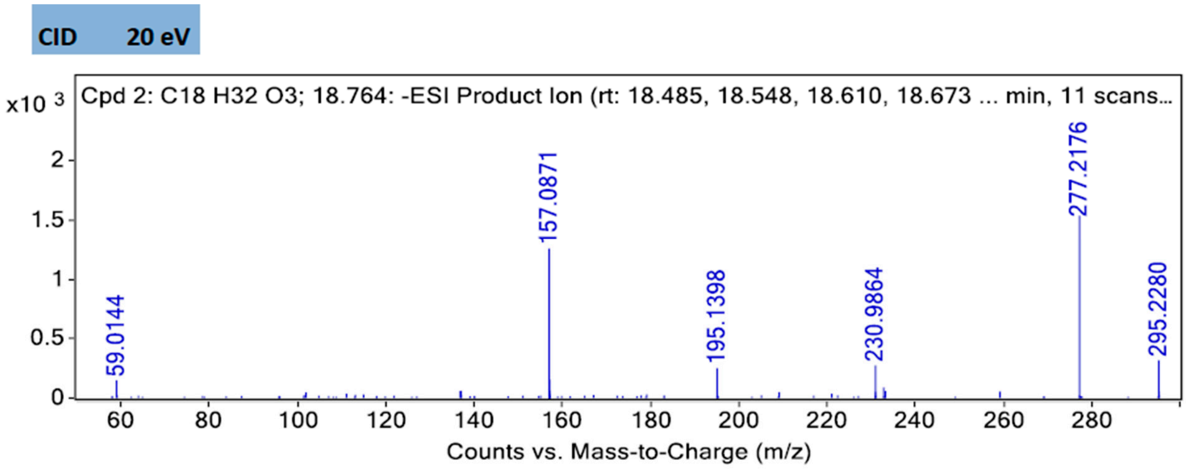

Figure S28. ESI-HRMS/MS spectrum of compound **1.1** isolated from *O. tomentosa*.

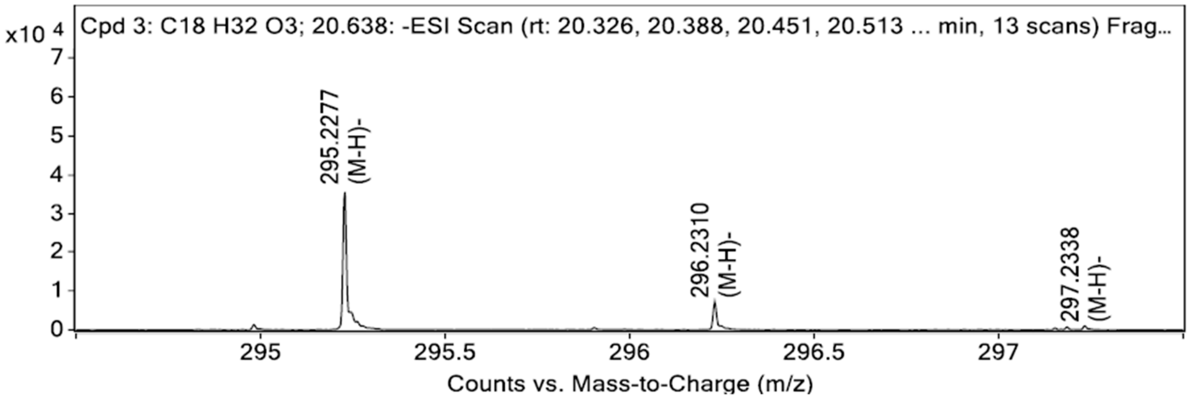

Figure S29. Isotope pattern of compound **1.2** isolated from *O. tomentosa*.

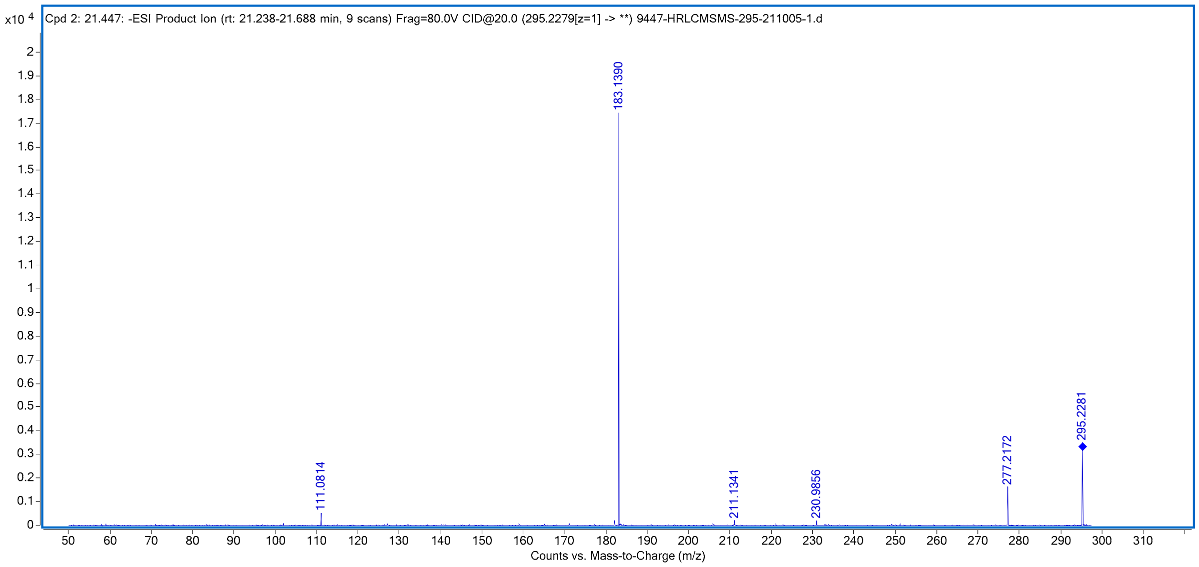

Figure S30. ESI-HRMS/MS spectrum of compound **1.2** isolated from *O. tomentosa*.

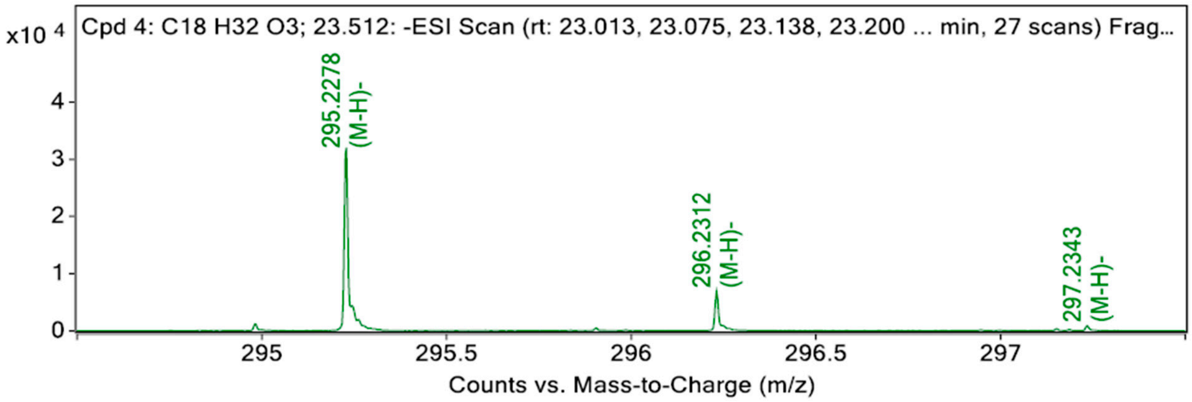

Figure S31. Isotope pattern of compound **1.3** isolated from *O. tomentosa*.

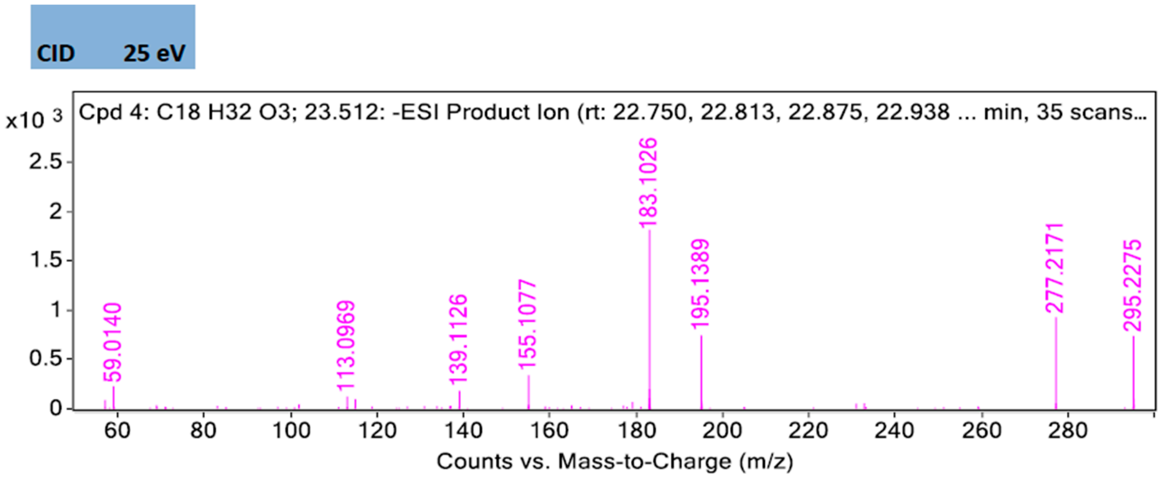

Figure S32. ESI-HRMS/MS spectrum of compound **1.3** isolated from *O. tomentosa*.

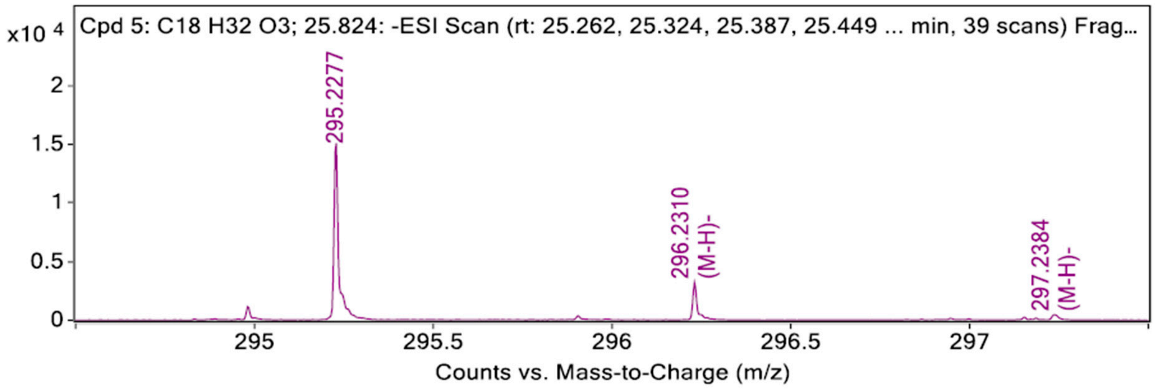

Figure S33. Isotope pattern of compound **1.4** isolated from *O. tomentosa*.

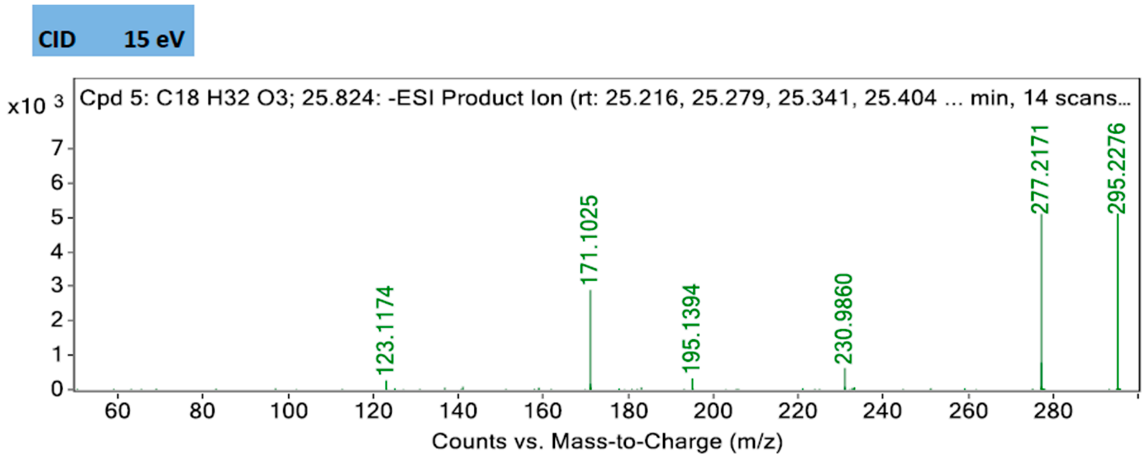

Figure S34. ESI-HRMS/MS spectrum of compound **1.4** isolated from *O. tomentosa*.

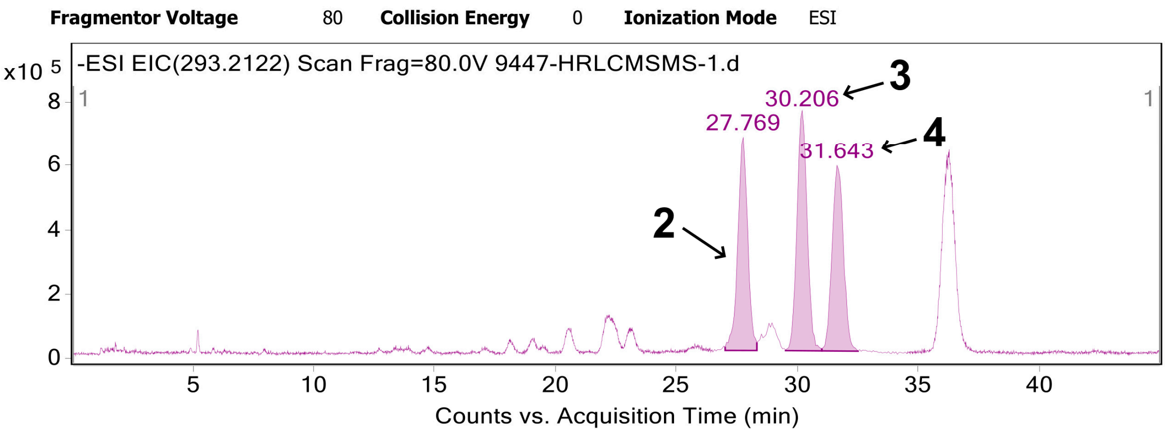

Figure S35. Extracted ion chromatogram (EIC) of compounds **2** to **4** isolated from *O. tomentosa*. The chromatographic system (column and solvents) used was as those described in Figure S26.

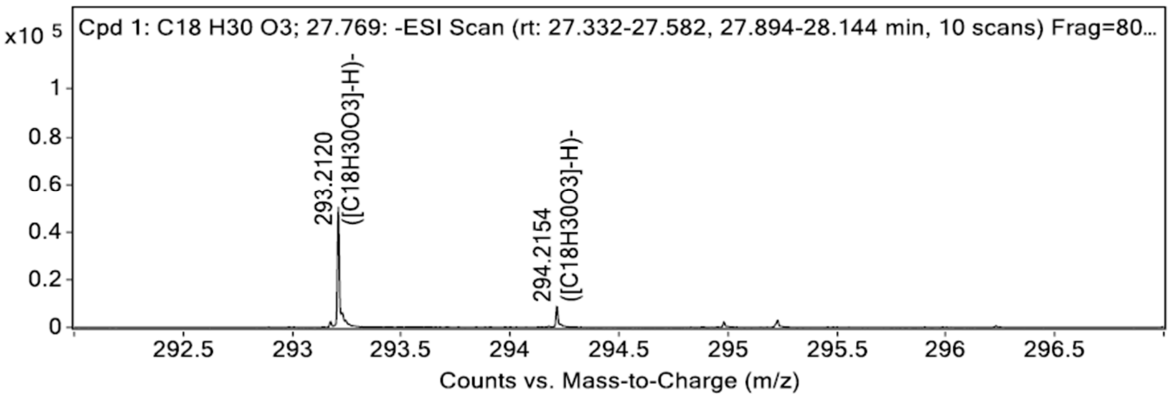

Figure S36. Isotope pattern of compound **2** isolated from *O. tomentosa*.

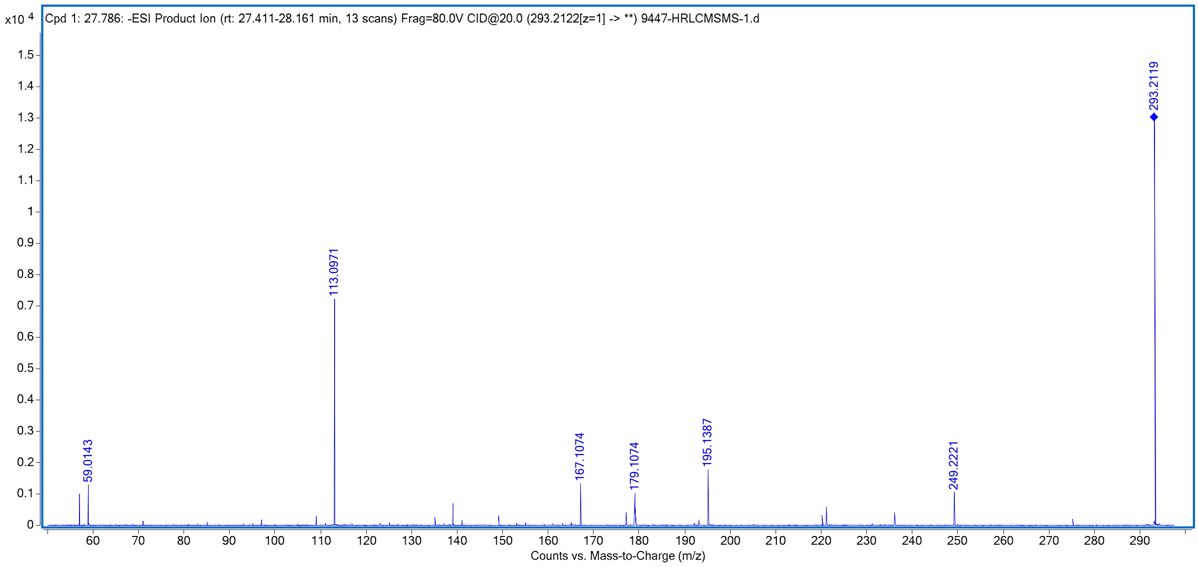

Figure S37. ESI-HRMS/MS spectrum of compound **2** isolated from *O. tomentosa*.

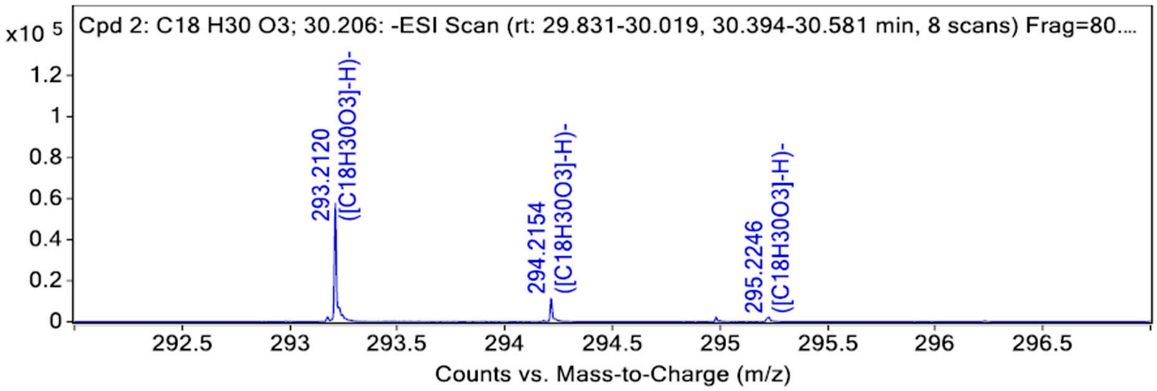

Figure S38. Isotope pattern of compound **3** isolated from *O. tomentosa*.

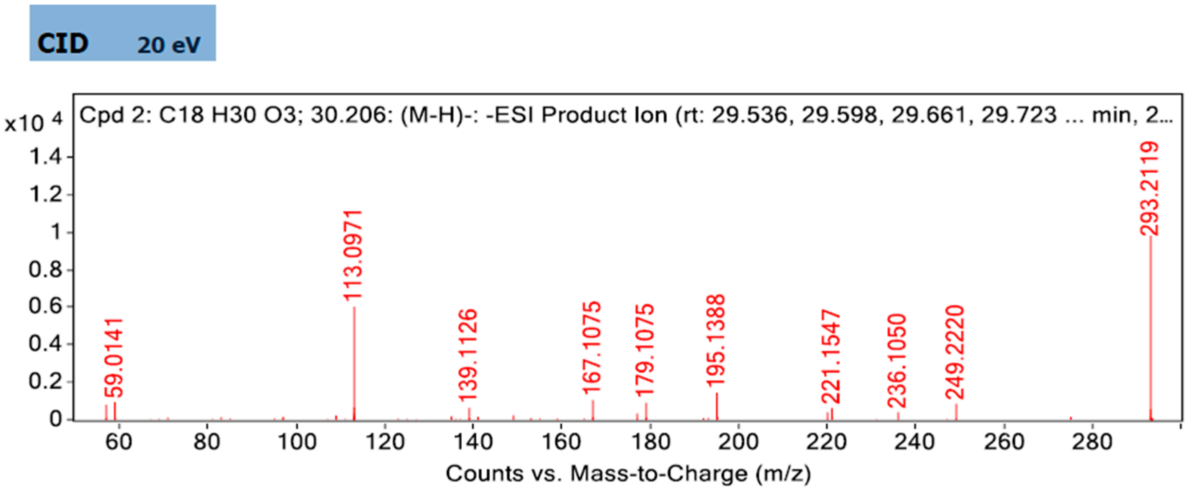

Figure S39. ESI-HRMS/MS spectrum of compound **3** isolated from *O. tomentosa*.

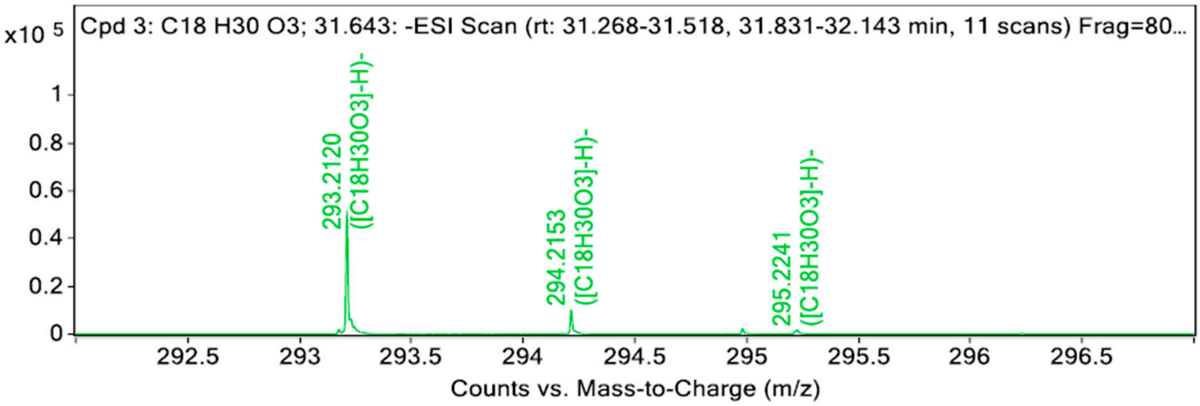

Figure S40. Isotope pattern of compound **4** isolated from *O. tomentosa*.

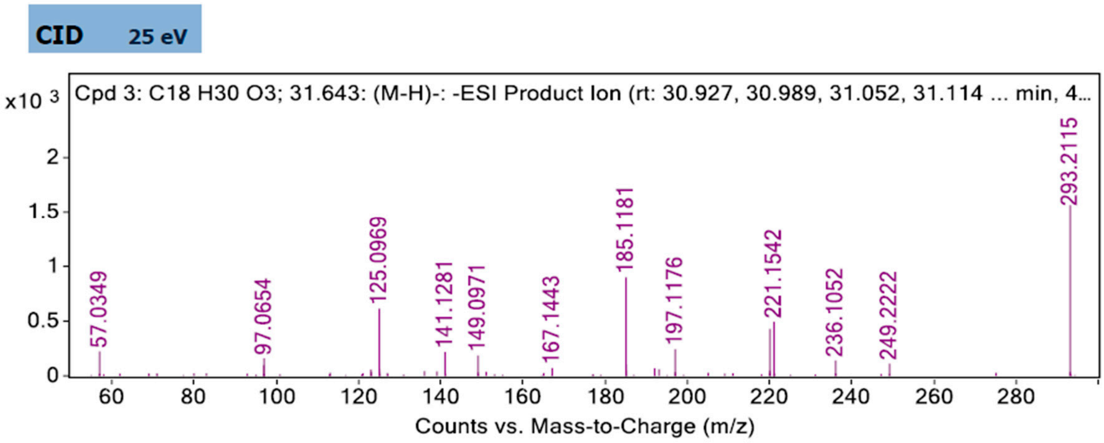

Figure S41. ESI-HRMS/MS spectrum of compound **4** isolated from *O. tomentosa*.

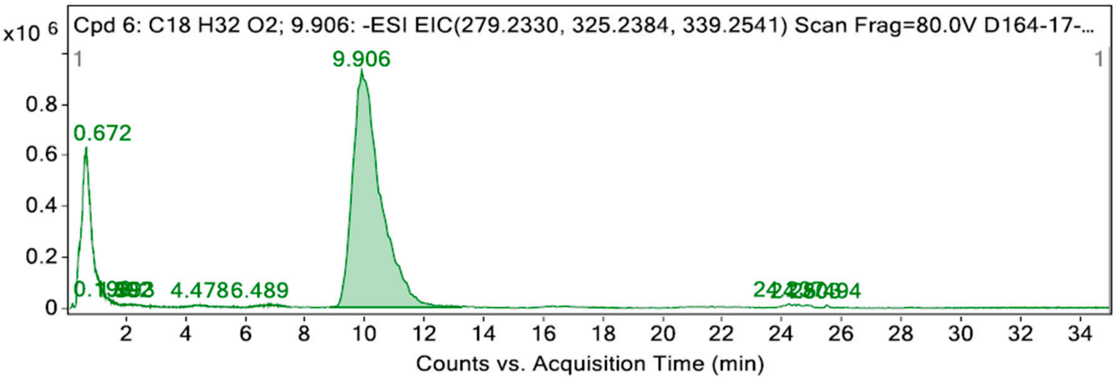

Figure S42. Extracted ion chromatogram (EIC) of compound **6** isolated from *O. tomentosa*. Analysis was done using an Agilent Infinity Lab Poroshell 120 EC-C18 column (2.1 mm × 50 mm × 2.7 µm) with a gradient mobile phase composed of an H_2O solution of 0.1% formic acid (solvent A) and CH_3CN containing 0.1% formic acid (solvent B); flow rate of 0.8 mL/min. The gradient elution program was set as follows: 0 min (52% B), 23 min (52% B), and 25 min (90% B).

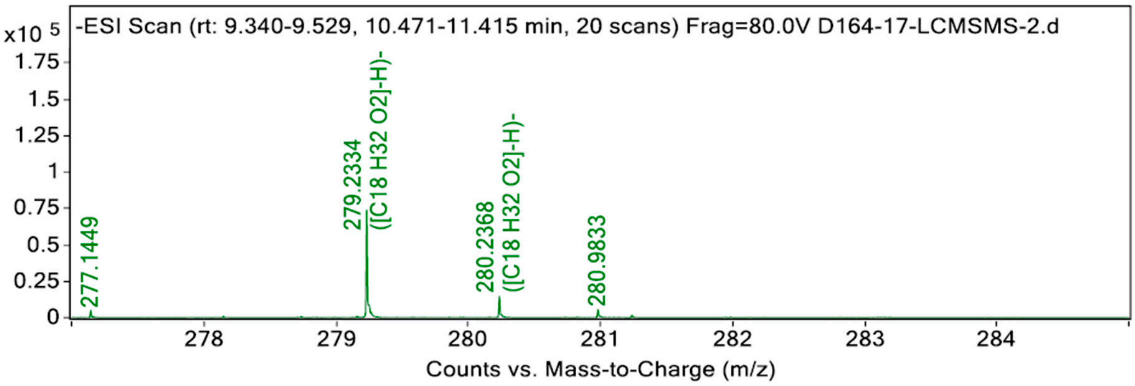

Figure S43. Isotope pattern of compound **6** isolated from *O. tomentosa*.

MSMS CID @ 30 eV

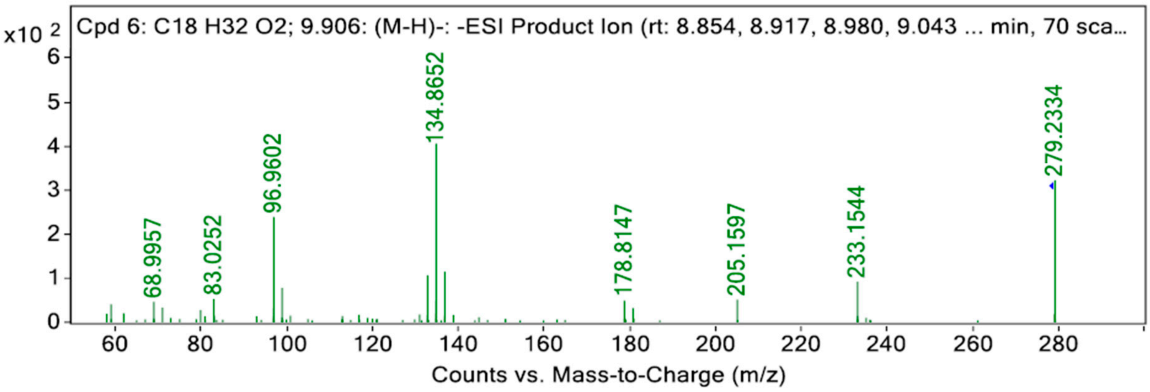

Figure S44. ESI-HRMS/MS spectrum of compound **6** isolated from *O. tomentosa*.

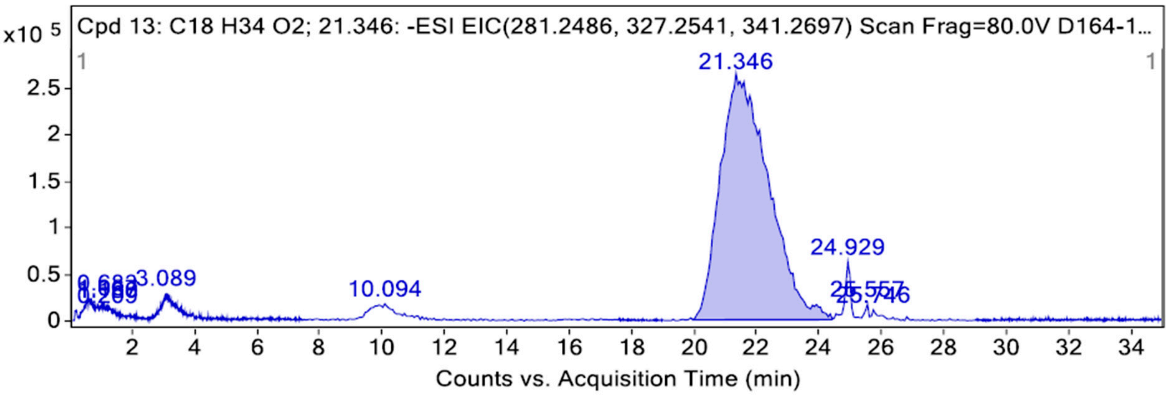

Figure S45. Extracted ion chromatogram (EIC) of compound **7** isolated from *O. tomentosa*. The chromatographic system (column and solvents) used was as those described in Figure S42.

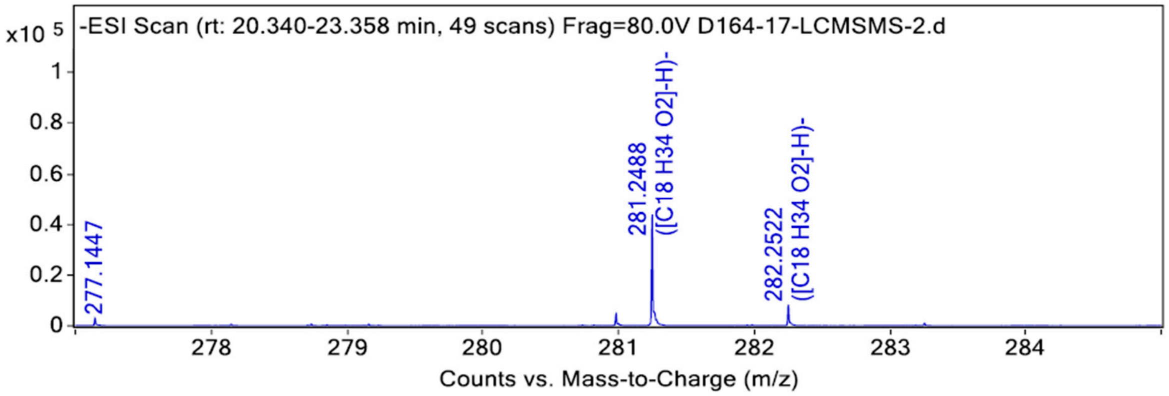

Figure S46. Isotope pattern of compound **7** isolated from *O. tomentosa*.

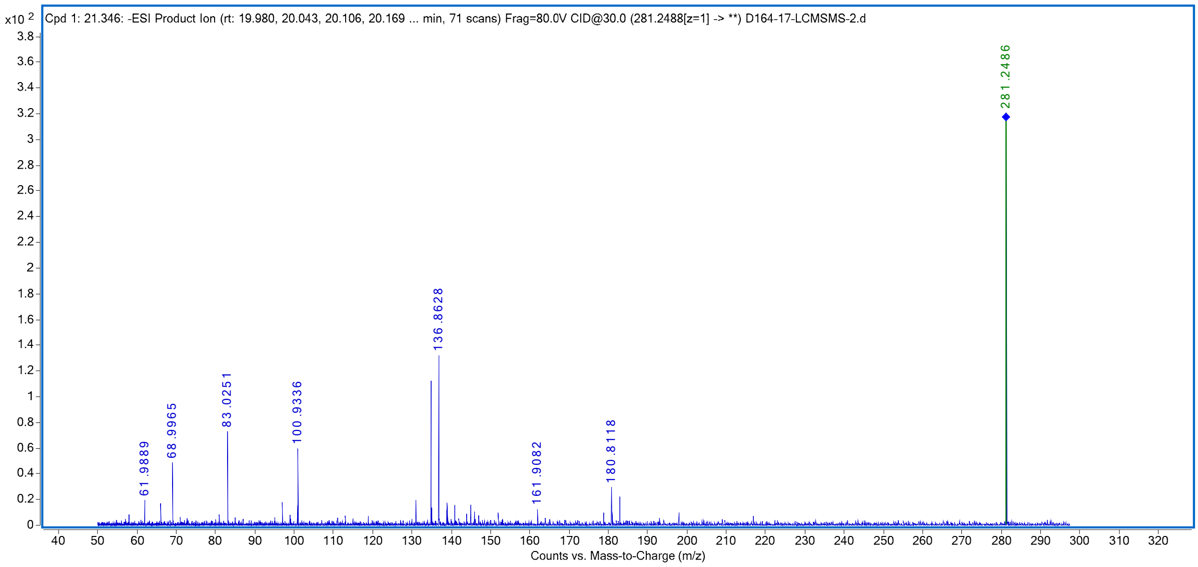

Figure S47. ESI-HRMS/MS spectrum of compound **7** isolated from *O. tomentosa*.

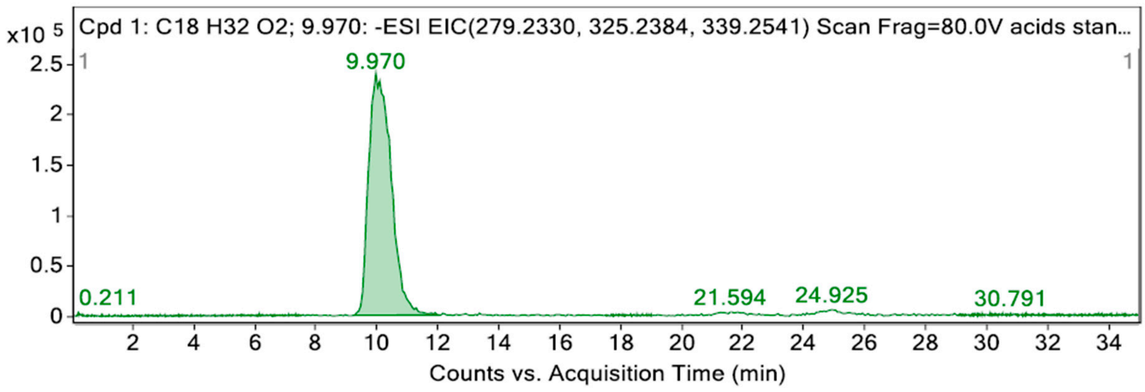

Figure S48. Extracted ion chromatogram (EIC) of pure standard linoleic acid. The chromatographic system (column and solvents) used was as those described in Figure S42.

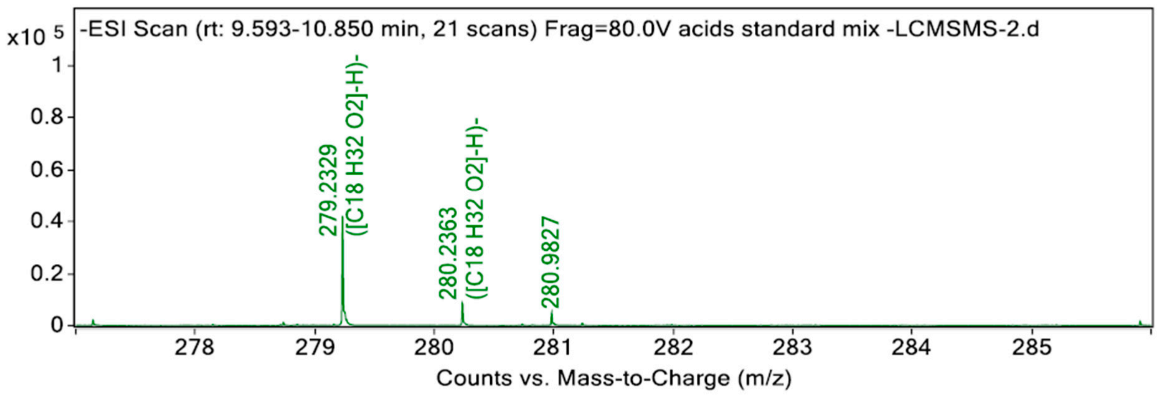

Figure S49. Isotope pattern of pure standard linoleic acid using ESI-HRMS.

MSMS CID @ 30 eV

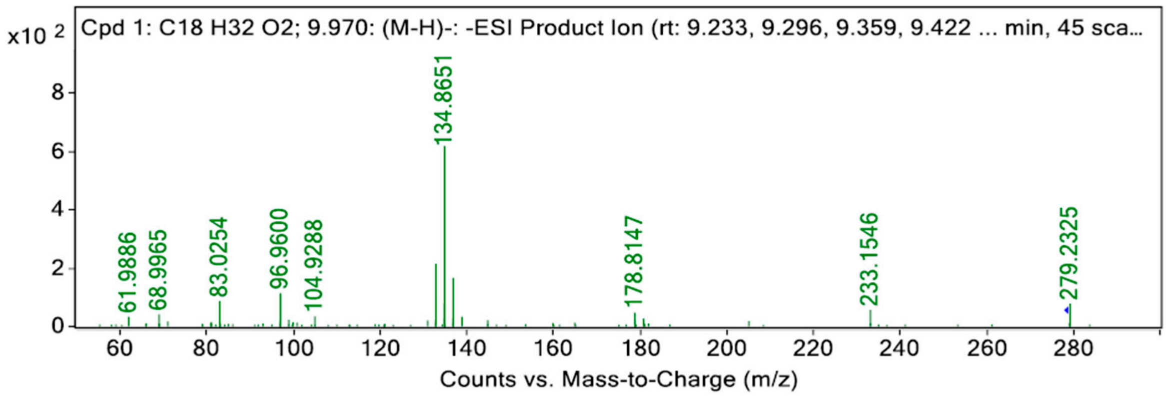

Figure S50. ESI-HRMS/MS spectrum of pure standard linoleic acid.

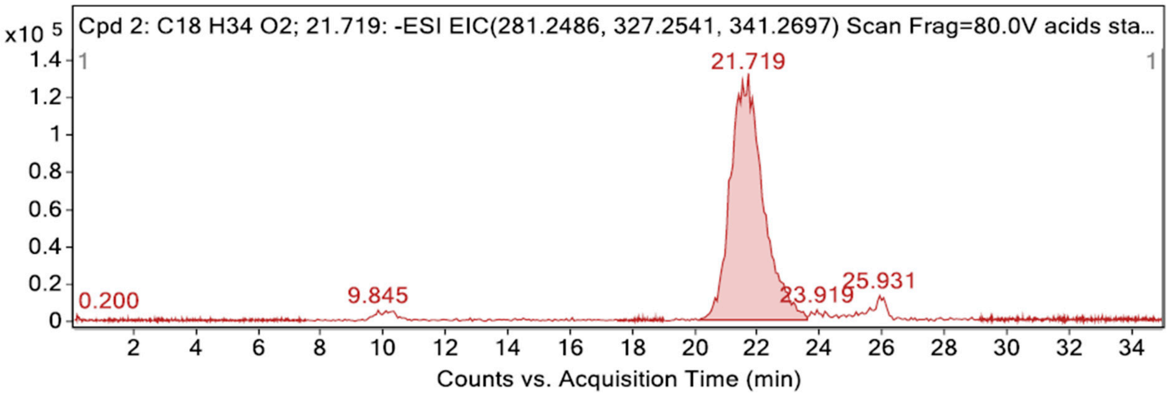

Figure S51. Extracted ion chromatogram (EIC) of pure standard oleic acid. The chromatographic system (column and solvents) used was as those described in Figure S42.

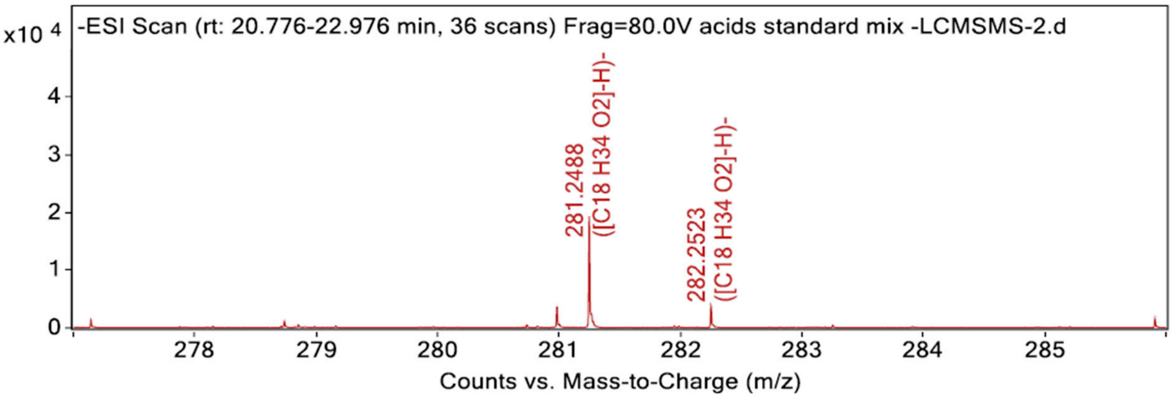

Figure S52. Isotope pattern of pure standard oleic acid using ESI-HRMS.

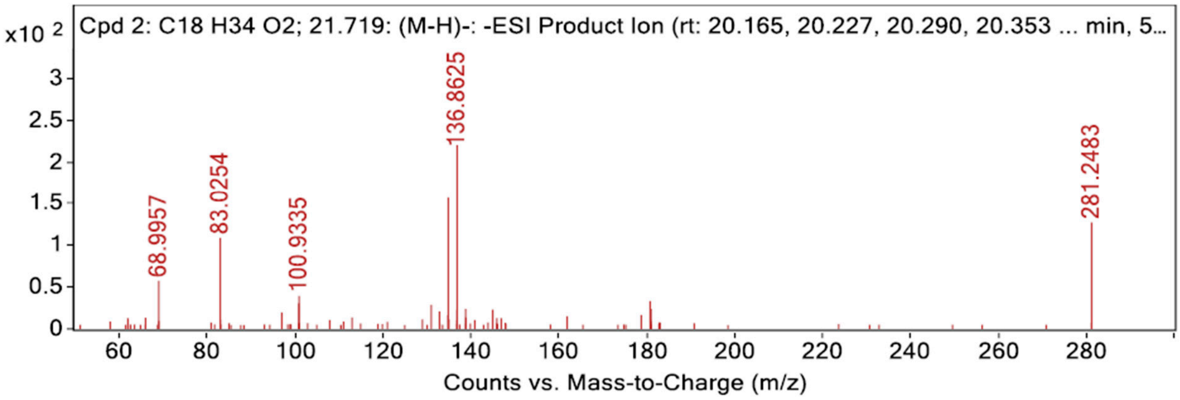

Figure S53. ESI-HRMS/MS spectrum of pure standard oleic acid.

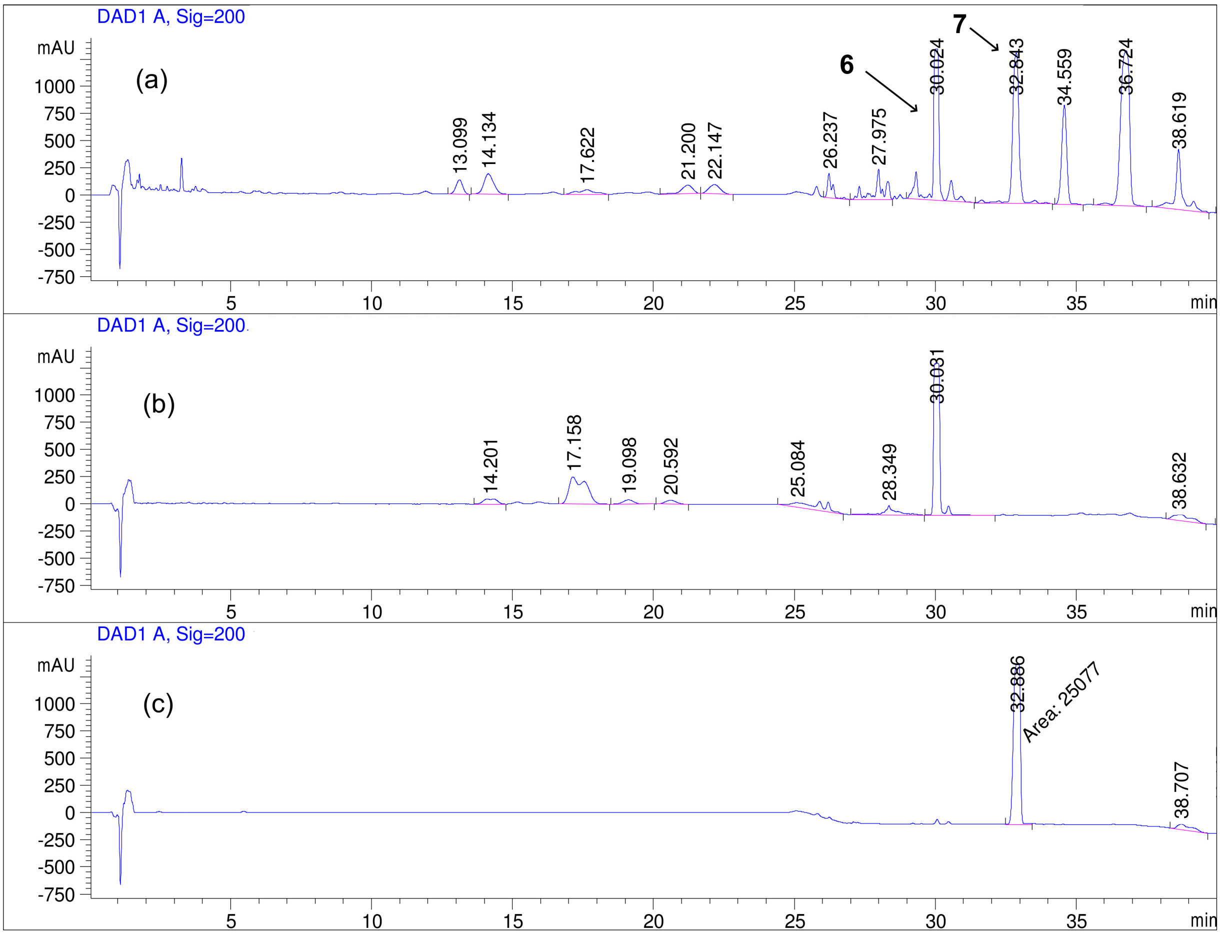

Figure S54. Comparative HPLC analyses of compounds **6** and **7** with their respective pure standards. (a) Post-Sephadex LH-20 sample from HEX layer; (b) linoleic acid; (c) oleic acid. The chromatographic system (column and solvents) used was as those described in Figure S12.

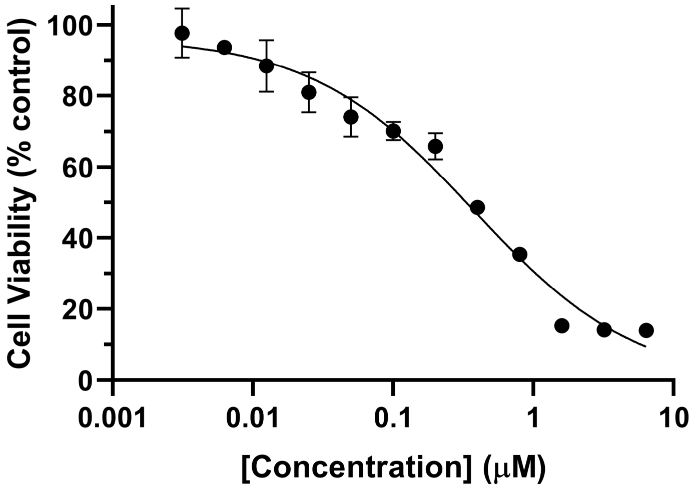

Figure S55. Effect of doxorubicin on HeLa cell viability. Cells were treated with different concentrations of doxorubicin (0.00312–6.4 μM) for 48 h, followed by cell viability assessment using the MTT assay. The result shown is representative data from three biological replicates (n = 3). Error bars indicate standard deviation (S.D.).

Table S1. Collection information for the four mushroom species.

Collection no.	Identity	Best GenBank match (% similarity/% coverage)	Collection site	Date
CL115	*Fomitopsis officinalis*	EU854436.1 (99.50%/83%)	Moore's Meadow, Prince George	September 2015
CL160	*Echinodontium tinctorium*	KF996511.1 (98.84%/95%)	Twin Falls Recreation Site, Smithers	September 2018
CL206	*Albatrellus flettii*	JF899544.1 (99.82%/100%)	Twin Falls Recreation Site, Smithers	August 2020
CL83	*Onnia tomentosa*	KF996517.1 (99.22%/81%)	John Prince Research Forest, Fort St. James	August 2015
CL312	*Onnia tomentosa*	KC152134.1 (99.41%/73%)	Greenway Trail, Prince George	August 2021

Table S2. The fatty acid composition in the four different mushroom species (µg/g).

Fatty acids	*F. officinalis*	*E. tinctorium*	*A. flettii*	*O. tomentosa*
C11:1	0.0	0.0	58.5	0.0
C12:0	168.9	46.3	46.0	78.2
C12:1	114.4	25.7	14.1	22.5
C14:0	70.5	26.1	107.4	104.4
C14:1	0.0	28.7	25.9	45.4
C15:0	70.2	93.2	645.0	332.6
C15:1	149.6	33.7	9.8	37.6
C16:0	1286.5	696.6	2462.7	3210.5
C16:1	58.4	98.9	266.3	161.5
C16:1-*trans*	55.6	43.3	78.5	41.8
C17:0	75.4	39.4	673.4	63.7
C17:1	0.0	52.4	0.0	0.0
C18:0	961.6	496.2	1131.3	987.0
C18:1 (oleic)	12180.3	1652.9	6900.0	5363.8
C18:1 (vaccenic)	555.6	132.7	678.3	342.3
C18:2n6	3651.0	755.9	15677.6	11012.4
C18:2n6-*trans*	0.0	21.6	24.0	28.7
C18:3n3	1.0	5.9	56.2	45.7
C18:3n6	86.0	21.4	25.3	24.2
C18:4n3	94.9	16.0	9.4	0.0
C19:1	0.0	8.1	35.6	0.0
C20:0	65.6	44.4	127.7	62.8
C20:1n11-*cis*	89.5	27.1	70.3	57.5
C20:1n15-*cis*	715.6	58.2	73.5	47.5
C20:2n6	64.6	17.9	346.8	71.8
C20:3n6	219.4	40.7	36.0	50.1

C20:4n6	65.7	65.5	31.9	48.9
C20:5n3	5.9	12.9	23.6	67.6
C22:0	83.4	51.7	284.4	74.6
C22:1	59.7	9.8	77.8	16.5
C22:4n6	70.3	0.0	0.0	0.0
C22:5n6	117.9	2.3	0.0	0.0
C22:6n3	155.3	108.2	167.4	96.2
C24:0	37.9	22.3	22.1	21.9
C24:1	0.0	0.0	61.8	190.0
Total Fatty Acid (as T.G.)	21330.7	4758.8	30307.6	22760.7
SFAs (μg)	2820.0	1516.1	5500.0	4935.6
MUFAs (μg)	13978.6	2174.4	8362.7	6338.9
PUFAs (μg)	4532.0	1068.3	16444.8	11486.2
Omega-3 Fatty Acids	257.1	143.0	267.8	220.8
Omega-6 Fatty Acids	4274.9	925.3	16177.0	11265.4
trans Fatty Acids	55.6	64.9	102.5	70.5
g of fatty acids/100g dried sample	2.1	0.5	3.0	2.3

SFAs: ∑saturated fatty acids; MUFAs: ∑monounsaturated fatty acids; PUFAs: ∑polyunsaturated fatty acids. Fatty acids at < 0.2% of the total fatty acid yield were excluded.

Table S3. The fatty acids composition in different *Inonotus* species.

**Fatty acids**	***I. hispidus*** [1]	***I. obliquus*** [2]	***I. radiatus*** [1]
C15:0	**6.4**	1.16	1.4
C16:0	1.8	**13.1**	**11.9**
C18:1	**27.9**	-	**39.6**
C18:2n6	3.2	**21.5**	**35.2**
C20:0	**47.9**	-	0.6

Bold font indicates the major fatty acids detected in the respective species.

References

1. Olennikov, D.N.; Sof'ya, V.A.; Tat'yana, A.P.; Borovski, G.B. Fatty acid composition of fourteen wood-decaying basidiomycete species growing in permafrost conditions. *Rec. Nat. Prod.* **2014**, *8*, 184.
2. Ayoub, N.; Lass, D.; Schultze, W. Volatile constituents of the medicinal fungus chaga *Inonotus obliquus* (Pers.: Fr.) Pilát (Aphyllophoromycetideae). *Int J Med Mushrooms* **2009**, *11*.
